# Exploring Genetic Diversity within *aus* Rice Germplasm: Insights into the Variations in Agro-morphological Traits

**DOI:** 10.1186/s12284-024-00700-4

**Published:** 2024-03-25

**Authors:** Puranjoy Sar, Sonal Gupta, Motilal Behera, Koushik Chakraborty, Umakanta Ngangkham, Bibhash Chandra Verma, Amrita Banerjee, Prashantkumar S. Hanjagi, Debarati Bhaduri, Sandip Shil, Jitendra Kumar, Nimai Prasad Mandal, Paresh Chandra Kole, Michael D. Purugganan, Somnath Roy

**Affiliations:** 1 Central Rainfed Upland Rice Research Station, ICAR-National Rice Research Institute, Hazaribag, Jharkhand 825 301 India; 2 Center for Genomics and Systems Biology, New York University, New York, NY USA; 3 Crop Physiology and Biochemistry Division, ICAR-National Rice Research Institute, Cuttack, Odisha 753 006 India; 4 Manipur Center, ICAR Research Complex for NEH Region, Imphal, Manipur, 795 004 India; 5 Crop Production Division, ICAR-National Rice Research Institute, Cuttack, Odisha 753 006 India; 6 Research Centre - Mohitnagar, ICAR-Central Plantation Crops Research Institute, Jalpaiguri, West Bengal 735 101 India; 7 Palli Siksha Bhavana (Institute of Agriculture), Visva-Bharati, Sriniketan, West Bengal 731236 India

**Keywords:** Rice, *aus* rice, GWAS, Yield, Agronomic Traits

## Abstract

The *aus* (*Oryza sativa* L.) varietal group comprises of aus, boro, ashina and rayada seasonal and/or field ecotypes, and exhibits unique stress tolerance traits, making it valuable for rice breeding. Despite its importance, the agro-morphological diversity and genetic control of yield traits in *aus* rice remain poorly understood. To address this knowledge gap, we investigated the genetic structure of 181 *aus* accessions using 399,115 SNP markers and evaluated them for 11 morpho-agronomic traits. Through genome-wide association studies (GWAS), we aimed to identify key loci controlling yield and plant architectural traits.

Our population genetic analysis unveiled six subpopulations with strong geographical patterns. Subpopulation-specific differences were observed in most phenotypic traits. Principal component analysis (PCA) of agronomic traits showed that principal component 1 (PC1) was primarily associated with panicle traits, plant height, and heading date, while PC2 and PC3 were linked to primary grain yield traits. GWAS using PC1 identified *OsSAC1* on Chromosome 7 as a significant gene influencing multiple agronomic traits. PC2-based GWAS highlighted the importance of *OsGLT1* and OsPUP4/ Big Grain 3 in determining grain yield. Haplotype analysis of these genes in the 3,000 Rice Genome Panel revealed distinct genetic variations in *aus* rice.

In summary, this study offers valuable insights into the genetic structure and phenotypic diversity of *aus* rice accessions. We have identified significant loci associated with essential agronomic traits, with *GLT1, PUP4*, and *SAC1* genes emerging as key players in yield determination.

**Supplementary Information:**

The online version contains supplementary material available at 10.1186/s12284-024-00700-4.

## Background

Asian cultivated rice (*Oryza sativa* L.) is a crucial staple food crop for more than one half of global population (Khush 2005). Being one of the earliest domesticated crops and a model organism, the phylogenetic and geographic origins of rice is well studied (Molina et al. 2011; Gross and Zhao 2014; Gutaker et al. 2020). Based on ecology, genetics and genomics, *O. sativa* is broadly classified into *indica, aus, temperate japonica, tropical japonica* and *aromatic* groups (Glaszmann 1987; Garris et al. 2005; Zhao et al. 2011; Wang et al. 2018). The *aus* group which was initially geographically assigned to South and West Asia (Glaszmann 1987), is now suggested to have originated from central India or Bangladesh based on comprehensive genomic data (Civáň et al. 2015). The *aus* group comprises of two seasonal ecotypes: aus and boro (Li et al. 2014; Alexandrov et al. 2015) distributed in both Bangladesh and India (Travis et al. 2015). The ‘boro’ indicates the cropping season spanning December to May, while ‘aus/ahu’ refers to April to August. Both these ecotypes have been traditionally selected to complete the life cycle in a short period and to have tolerance to abiotic stresses like drought, cold and heat. Furthermore, the *aus* group also includes deep-water cultivars (referred as ‘ashina’ in Glaszmann 1987) from Assam and Bangladesh, as well as the ‘rayada’ cultivars originating from a small geographical area along the Madhumati river in Bangladesh (Rubaiyath Bin Rahman and Zhang 2013). All these *aus* cultivar types have been categorized as circum-*aus* in Wang et al. (2018). In this paper, we have the term ‘*aus*’ in italic font to referrer to the genetic group, while ‘aus’ in non-italic font refers to seasonal ecotype.

*Aus* cultivars group has immense potential for utilization in breeding due to its tolerance many abiotic stress factors. While stress tolerance studies have traditionally focused on the Japonica variety Nipponbare, which benefits from available genetic resources and a reference genome, recent advancements in de novo reference genomes for *aus* cultivars like N22 and Kasalath offer valuable insights into *aus*-specific genes and pathways. Genetic analysis of the 3,000 Rice Genome Project (3K-RGP) accessions (Li et al. 2014) revealed a greater abundance of ‘private’ alleles in *aus* compared to other rice groups, particularly around major domestication genes like *Sh4, sd1, Wx*, and *Rc* that control traits such as grain shattering, semi-dwarf height, grain amylose content, and pericarp colour, respectively (Wang et al. 2018) Moreover, considerable population structural diversity within *aus* (Norton et al. 2018) can be exploited suitably for rice improvement.

A range of crucial stress tolerance genes such as *OsSub1, SNORKELs, OsPSTOL1*, and *Dro1*, were first reported in *aus* genotypes (Bin Rahman and Zhang 2018). Notably, these genes are absent from the Nipponbare reference sequence. This underlines the potential of *aus* germplasm for unveiling novel allelic variations associated with crucial agronomic traits to safeguard rice production from the progressive changes in global climate causing frequent extreme weather events like drought, flooding, and high temperature. With high-quality SNP data accessible for diverse rice germplasm panels like BAAP and 3 K-RGP (Rice SNP-seek database; https://snp-seek.irri.org), genome-wide association studies (GWAS) offers a compelling approach to uncover natural variations pertaining to agronomic, grain quality, and stress tolerance traits (Norton et al. 2018; Bhandari et al. 2020).

To date, the morphological diversity of *aus* rice has not been evaluated on a global scale. Therefore, it is interesting to explore the genetic basis of the phenotypic diversity which will help better utilization of *aus* germplasm in rice breeding. In this study we evaluated 181 *aus* rice from the 3 K-RGP panel for 42 agro-morphological traits with following objectives: (i) to understand how well the agro-morphological diversity correlates with the population genetic structure, and how it relates to the origin and distribution of *aus* cultivars, (ii) to determine the genetic factors associated with the agronomical features of *aus* rice using GWAS, and compare those with earlier reports on diverse rice germplasm.

## Materials and Methods

### Plant Materials and Growth Conditions

**Fig. 1:**
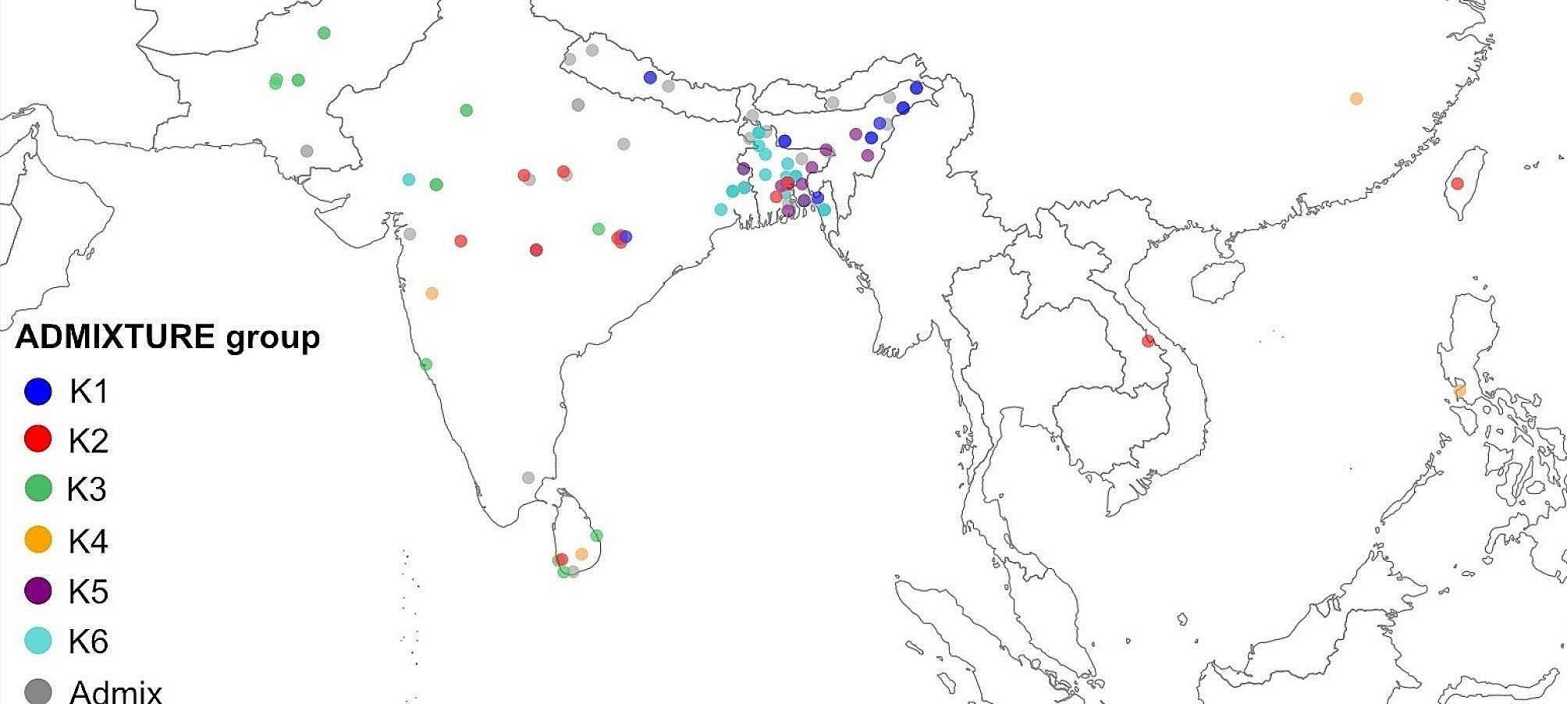
**Geographical distribution of*aus*rice accessions used in the study.** A few accessions from other parts of the world have not been shown here. Each point indicates the geolocation of the cultivar as given in https://www.genesys-pgr.org. The colour of the points indicates to which *aus* sub-group the accession belongs (see Fig. 2) based on population structure analysis at *K =* 6

A total of 181 *aus* rice accessions from the 3000 Rice Genome Project (3 K-RGP) (Li et al. 2014) were included in the study (Dataset S1). Originally, there were 214 *aus* accessions in the 3 K-RGP, but we could obtain seeds of 181 accessions from the IRRI genebank. The passport data on the origin of accessions were obtained from Genesys (https://www.genesys-pgr.org/). The geographic distribution of the *aus* accessions is shown in Fig. 1.

During the wet season (June-November) of 2020, 181 *aus* accessions were cultivated at Hazaribag, Jharkhand (23.9596 °N, 85.3739 °E, 600 m) under rainfed conditions. Each accession was grown under puddled transplanted conditions in a 2.5 m × 1.5 m plot with a spacing of 20 cm (row-to-row) and 15 cm (plant-to-plant) following an augmented block design. Three check varieties: Vandana, Sahbhagi Dhan and IR64 were included. Standard production practices were followed to manage the crop.

### SNP Genotypic Data and Filtering

The genotypic data for the 181 accessions were obtained from the data repository of 3 K-RGP (https://snp-seek.irri.org/). The raw genotypic data we considered from 3 K-RP Base SNP dataset which contained 18,128,777 SNPs. The Base SNP dataset was originally created from ~ 29 million biallelic SNPs by removing SNPs with excess of heterozygous calls. The marker set was then filtered using nucleotide variation missing rate < 0.20 and a minor allele frequency (MAF) > 0.01 using PLINK (Purcell et al. 2007).

### Population Structure

The MAF-filtered data was further thinned by applying a two-step LD pruning using PLINK (“indep-pairwise 10 kb 1 0.8” and “indep-pairwise 50 1 0.8”). The resulting set of 399, 115 SNP (referred to as 399 K set) was then used for population structure analysis.

The population structure of 181 *aus* genotypes was assessed using ADMIXTURE v. 1.3.0 (Alexander et al. 2009). Sub-populations from *K* = 1 to 8 were tested. Since the cross-validation error barely differed between *K* values beyond *K* = 6, we defined *K* = 6 as optimal clusters in *aus* germplasm (Fig. S1). An 80% threshold of cluster membership was used to assign cultivars into population sub-groups. Furthermore, population structure analysis of *aus* genotypes from India (71 accessions) and Bangladesh (76 accessions) was assessed independently using similar criteria. The Principal component analysis (PCA) for all accessions was done using TASSEL5 (Bradbury et al. 2007). A neighbour-joining tree was built by calculating the pairwise genetic distances between samples using the VCF2Dis software (https://github.com/BGI-shenzhen/VCF2Dis). From the genetic distance matrix, a neighbor-joining tree was built using the programme FastME (Lefort et al. 2015).

### Phenotype Data Recording and Statistical Analysis

Forty-two phenotypic traits comprising of 11 agronomical and 30 qualitative morphological variables, were evaluated following standard procedure (IRRI 2013); for details see Dataset S2). Data on agronomical variables were recorded from 15 randomly chosen plants in each plot (excluding the border rows) and grain yield data was recorded from plot yield as yield per m^2^ after threshing and drying the seed to around 14% moisture content.

The phenotyping data for agronomic (quantitative) variables were analyzed using an augmented block design with the R package augmentedRCBD (R Core Team 2021). Adjusted means, range, skewness, kurtosis, coefficient of variation (CV), genetic coefficient of variation (GCV), phenotypic coefficient of variation (PCV), broad sense heritability (h^BS^) and frequency distribution were calculated.

The 181 *aus* accessions were classified into seven genetic clusters: *K*1 to *K*6, and ‘admix’, based on Admixture analysis results. A univariate analysis using a general linear model for quantitative traits was performed for seven genetic cluster in SPSS Statistics v.21 (IBM, Armonk, NY). The ANOVA was calculated to find the significance of difference among the groups means. In addition, the comparison of means within each group was carried out using Tukey’s test at *P* < 0.05.

To investigate the relationships among agronomical variables and the factors underlying the trait variation, a principal component analysis (PCA) was carried out for 11 traits using GraphPad Prism v. 9.0. The variables were standardized to have a mean of 0 and standard deviation (SD) of 1 before analysis, and the principal components (PCs) were selected based on parallel analysis which performs 1000 Monte Carlo simulations on “random data” of equal dimension to the input data, and finally selects PCs with eigen values greater than those from the simulations at 95% percentile level.

### Genome-wide Association Analysis

The three principal components (PCs) obtained from PCA of 11 agronomical traits, along with the log_10_-transformed values of individual traits were included in GWAS (Dataset S3). We used a separate larger set of 458, 615 SNPs (referred as 458 K set) of 181 *aus* genotypes which was filtered from the base SNP set using the criteria: missingness (0.25) and MAF (0.05) and LD pruning (indep-pairwise 2 kb 1 0.8). The Fixed and Random Model Circulating Probability Unification (FarmCPU) model (Liu et al. 2016) in the R package of Genomic Association and Prediction Integrated Tool (GAPIT) (Wang and Zhang 2021) was used for GWAS analysis. The FarmCPU model is a multi-locus linear mixed model (MLMM) that improves statistical power and reduces both false positives and false negatives (Liu et al. 2016; Kaler et al. 2019). Population structure was accounted for by using a kinship matrix to reduce the occurrence of false positives and spurious associations. Quantile–quantile (Q-Q) plots of the estimated and observed P-values for marker–trait associations were generated to evaluate the model fit.

The critical *P*-value for explaining a significantly associated marker was the rather conservative Bonferroni correction, calculated by the–log10(p-value of 0.05/ΣSNPs), which corresponds to -log_10_(0.05/458,615) = 6.96. The percentage of total phenotypic variance (PVE) explained by significant MTAs was generated in GAPIT. The PVE of the markers is calculated in GAPIT as their corresponding variance divided by the total variance, which is the sum of residual variance and the variance of the associated markers, calculated using the R/lme4 package.

### Linkage Disequilibrium and Prediction of Candidate Genes

Linkage disequilibrium (LD) decay was measured by correlation coefficients (*r*2) for all pairs of SNPs with a sliding window approach with the following parameters: -MaxDist 500-MAF 0.05-Het 0.88-Miss 0.999 using PopLDdecay v3.27 (Zhang et al. 2019). The LD decay distance was determined when the LD *r*^2^ fell to 0.1. Considering the LD decay distance, we defined the interval of significantly associated SNP(s) ± LD decay distance as QTL regions.

The identified QTL regions covered by significant SNPs were searched for candidate genes or QTLs using the Rice SNP seek database (https://snp-seek.irri.org/_jbrowse.) which integrates various databases like QTARO, Oryzabase and MSU databases. For trait-associated SNPs, contingency tables between SNP alleles and phenotype were made and visually inspected to examine the associations.

The LDBlockshow (Dong et al. 2020) was used to estimate the local LD blocks within the QTL/ gene. Gene haplotype analysis was performed using all SNPs within the coding sequence region ignoring the synonymous SNPs. Haplotype analyses were done for the *aus* germplasm as well as the 3,020 accessions in the 3 K-RG using the Rice SNP-Seek database. Significant phenotypic differences among the haplotypes were determined using Tukey’s multiple comparisons test in one-way ANOVA using GraphPad Prism v. 9.0.

## Results

### Genetic Structure and Subgroupings Within *aus* rice

**Fig. 2:**
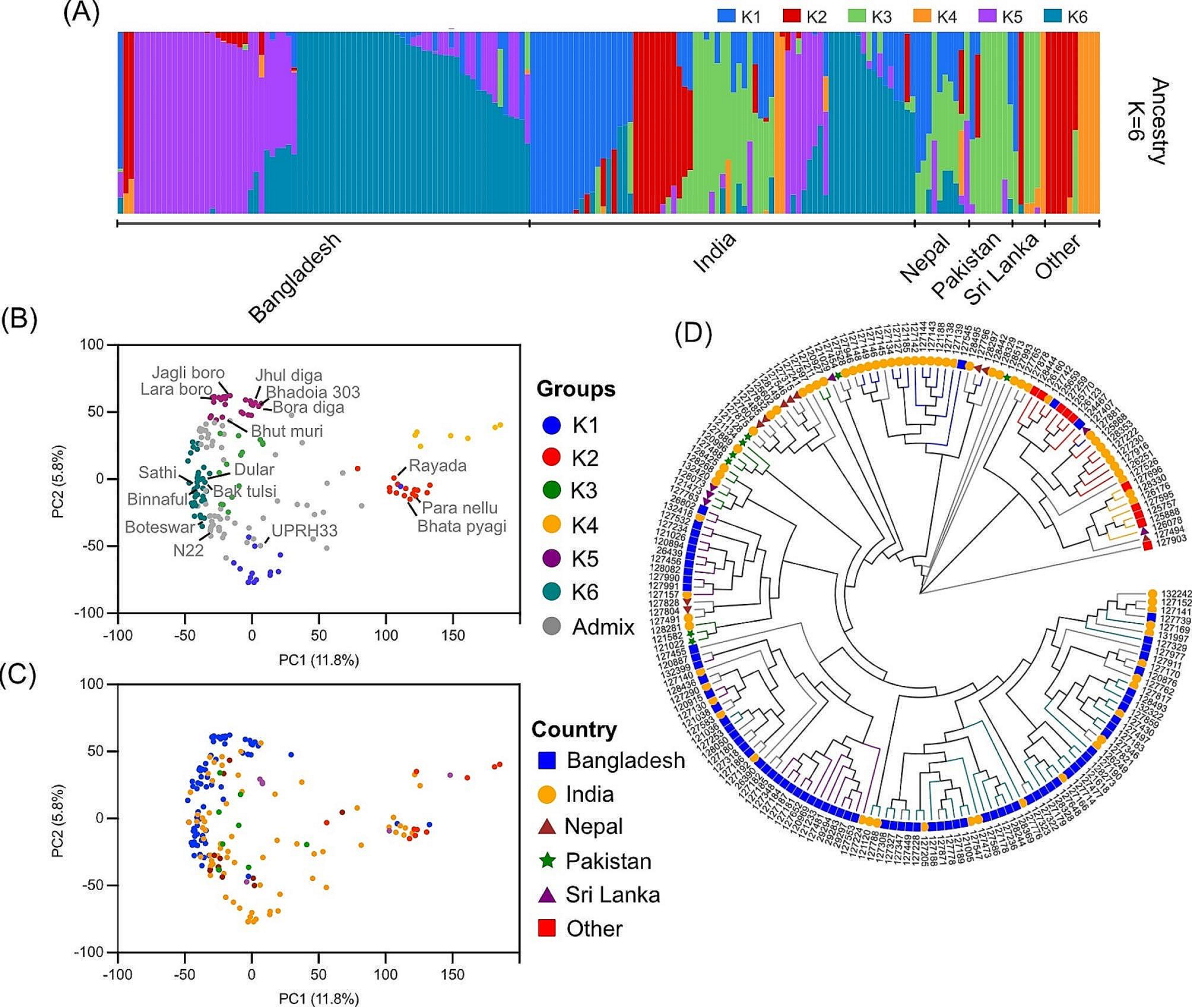
**Population structure of*aus*rice.A** The plot of ADMIXTURE subpopulation membership coefficients at *K* = 6. The cross-validation error values at different *K* indicates *K* = 6 as the most ideal sub-groups, **B** Biplot of first two PCA axes of 181 *aus* accessions colour coded according to the ADMIXTURE classification at *K* = 6, **C** Biplot of first two PCA axes of 181 *aus* accessions colour coded according to their geographical association. **D** NJ tree based on pairwise genetic distance. The accessions were colour coded according to the ADMIXTURE grouping (branch colour) as well as geographic origin as given in B and C

The patterns of the genetic structure of 181 *aus* accessions were analyzed using the 399 K SNP set. Using the cross-validation error values generated in ADMIXTURE by varying sub-groupings (*K*) from 1 to 8 (Fig. S1A), we found six subgroups (at ≥ 80% cut-off) designated as K1 to K6 (Fig. 2A). At *K* = 6, the *aus* germplasm from Bangladesh is mostly comprised of two clusters, whereas, the Indian germplasm exhibited a richer diversity with five clusters (Fig S1B-C; Dataset S1). The distinctness of six *aus* subgroups is also apparent in the PCA (Fig. 2B). To check the genetic differentiation of aus and boro ecotypes, we examined the clustering at *K* = 2. Interestingly, at this level, aus and boro ecotypes appeared to be genetically close as both were included in the same cluster (Dataset S1). However, at *K* = 6, the boro cultivars showed some degree of differentiation and all are grouped under K5 (Fig. 2B).

Genetic distance-based analysis revealed considerable geographical structuring (Figs. 1 and 2C and D). First, the genetic groups identified through ADMIXTURE analysis seem to be clustering geographically well. K1, K5 and K6 are close to each other on the map and these populations have low *F*_*ST*_ values (Table S1). K1 is mostly (87%) of Indian origin, while K5 and K6 are predominantly (86% and 75%, respectively) originating from Bangladesh. Overall, Among the subgroups, K3 and K6 are the closest (*F*_*ST*_ = 0.162), while K2 and K5 are the most distant (*F*_*ST*_ = 0.518).

Interestingly, although K3 has low overall *F*_*ST*_ with K1, K5 and K6, yet is geographically distant (located in North West India, Pakistan and Sri Lanka). As K3 included many early maturing drought-tolerant accessions (assessed in our separate study), it is possible that this groups was further extended geographically for their drought tolerance. The K2 represented by the rayada cultivar from Bangladesh along with the accessions from central India, Sri Lanka and the countries outside the Indian subcontinent had greater genetic distance than all other *aus* clusters. This is consistent with the previous reports that rayada cultivars are genetically distinct from most other *aus* cultivars (Wang et al. 2014). Overall, the geographical distribution and selection of accessions for specific ecologies seem to have played crucial roles in shaping the population structure of *aus* rice.

### Overall Agro-morphological Variability of *aus* rice

The analysis of agronomic traits revealed high phenotypic variability within *aus* population (Table S2; Fig. S2). Deviations from the normality have been observed from most of the traits except 1000-grain weight and grain yield plot^− 1^. Overall, as compared to check varieties, the *aus* genotypes exhibited early heading, taller plant height, fewer tiller, longer flag leaves, lesser grain weight, as well as lower yield and harvest index. Accessions such as P335 (342), Vaikatharyan (305), ARC 10,100, Begum, AUS177, Han Nuo, Jashure aus, Malagkit, and ARC13276 recorded higher (> 200) grains panicle^− 1^. Although a single accession, AUS177, showed higher yield than the best check Sahbhagi dhan (1375.2 g), there were ten other accessions which showed a higher yield than the second best check i.e., IR64 (1200.59 g). Noteworthy accessions with higher yield and harvest index were R762, PR106, Rantnagiri 45 − 2, Jabor sail, I Kung Pao, N22, NCS 840, Bhut muri, Herath banda, and Narikel badi. The correlation among the traits in *aus* germplasm is shown in (Fig. S3). Largely, the late maturing genotypes seems to have greater values of plant height, panicle length, spikelet number, panicle weight and yield.

### Phenotypic Diversity Among *aus* Genetic Subgroups

**Fig. 3:**
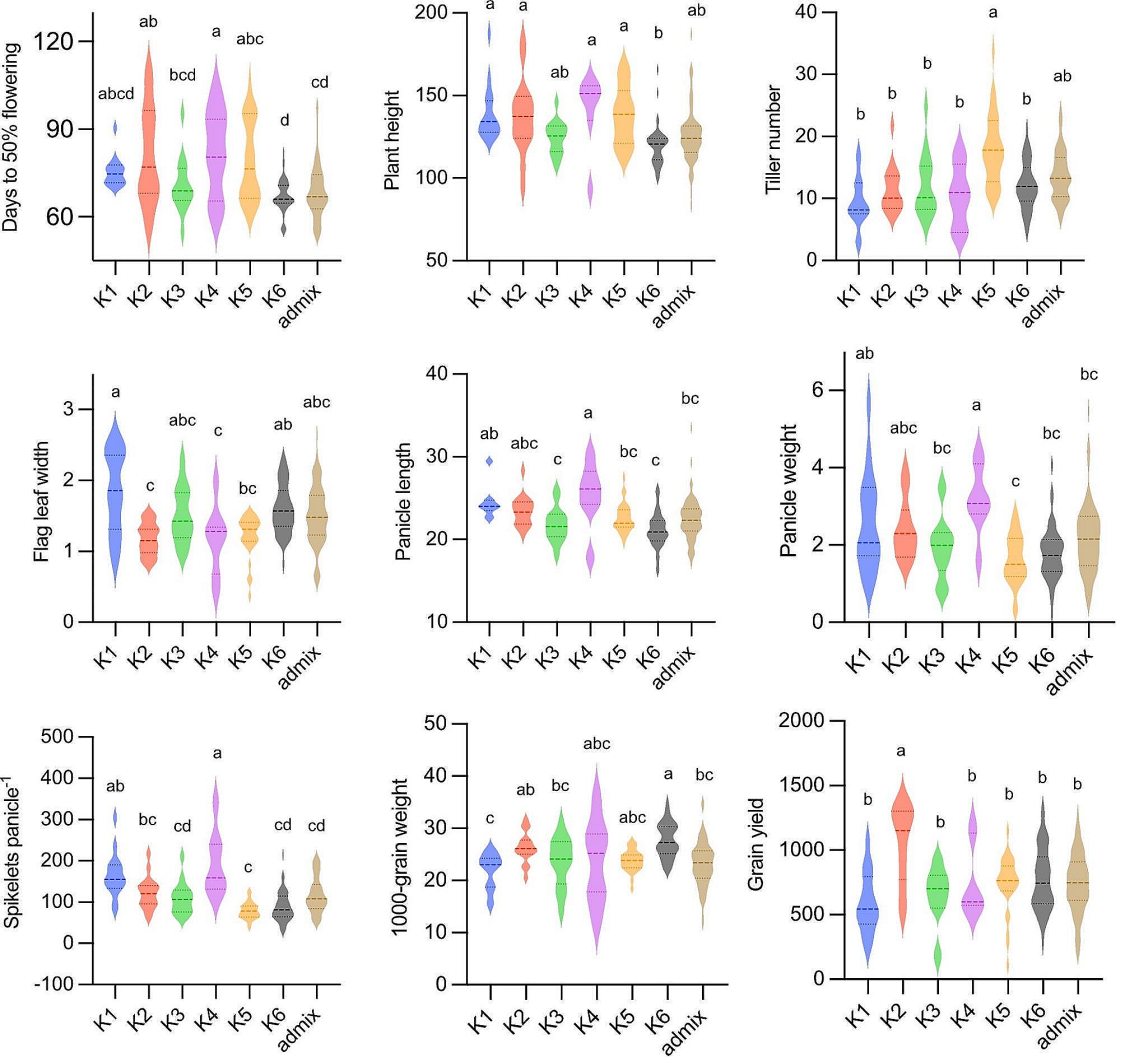
**Differences for quantitative morphological traits among the*aus*subgroups.** Multiple comparison of Trait means of subgroups was done using the Tukey’s HSD test. The trait means of subgroups with the same letters above the violin plots are not significantly different. The traits not significantly differing among the subgroups are not shown here

We have analyzed the agro-morphological differences between the *aus* subgroups identified through ADMIXTURE analysis. There are significant differences for most of the agronomical traits except flag leaf length and harvest index (Fig. 3; Dataset S4). However, for 30 qualitative traits, we have found considerable overlapping among the subgroups for many of the traits (Fig. S4-S5).

The K1, represented mostly by Assam rice accessions, is poor yielding due to lower tillering despite bearing higher number of spikelets per panicle. K2, the genetically most distinct subgroup, has the highest yield potential resulting from heavier panicles with higher spikelet numbers. K3 subgroup is early maturing with a moderate yield level. The accessions belonging to K4 have low yield potential largely due to delayed flowering and tall plants, despite having the most desirable panicle traits. The boro and deep-water cultivars grouped in K5 showed wide variation for days to heading, taller plants, high tillering ability, and inferior panicle traits resulting in a moderate level of grain yield. The K6 included drought tolerant cultivars having earliest to flowering, shortest plant height, and smaller panicles with lesser but heavier grains, resulting in a moderately high yield level (Fig. 3). Overall, the phenotypic variability of the genetic groups showed substantial linkage with their growing ecology or geographical distribution.

The frequency distribution of 30 qualitative morphological variables within different *aus* subgroups indicated considerable overlapping among the genetic groups for most of the traits. Purple colouration of basal leaf sheaths, internodes, apiculi, and auricles occurred in higher frequency in K6 and K5, and less in K4. The accessions of K5 and K6 subgroups showed spreading type plant architecture in higher frequency. The presence of awnned cultivars was noted in all subgroups, but occurred in higher frequency in K5, K3 and K4. Red seed coat colour is most frequent (90%) in K6. The glutinous endosperm was mostly frequent in the accessions of K1 and K2 which explained by their geographical distribution in Northeastern India and southeast Asian countries where glutinous rice is preferred.

**Fig. 4:**
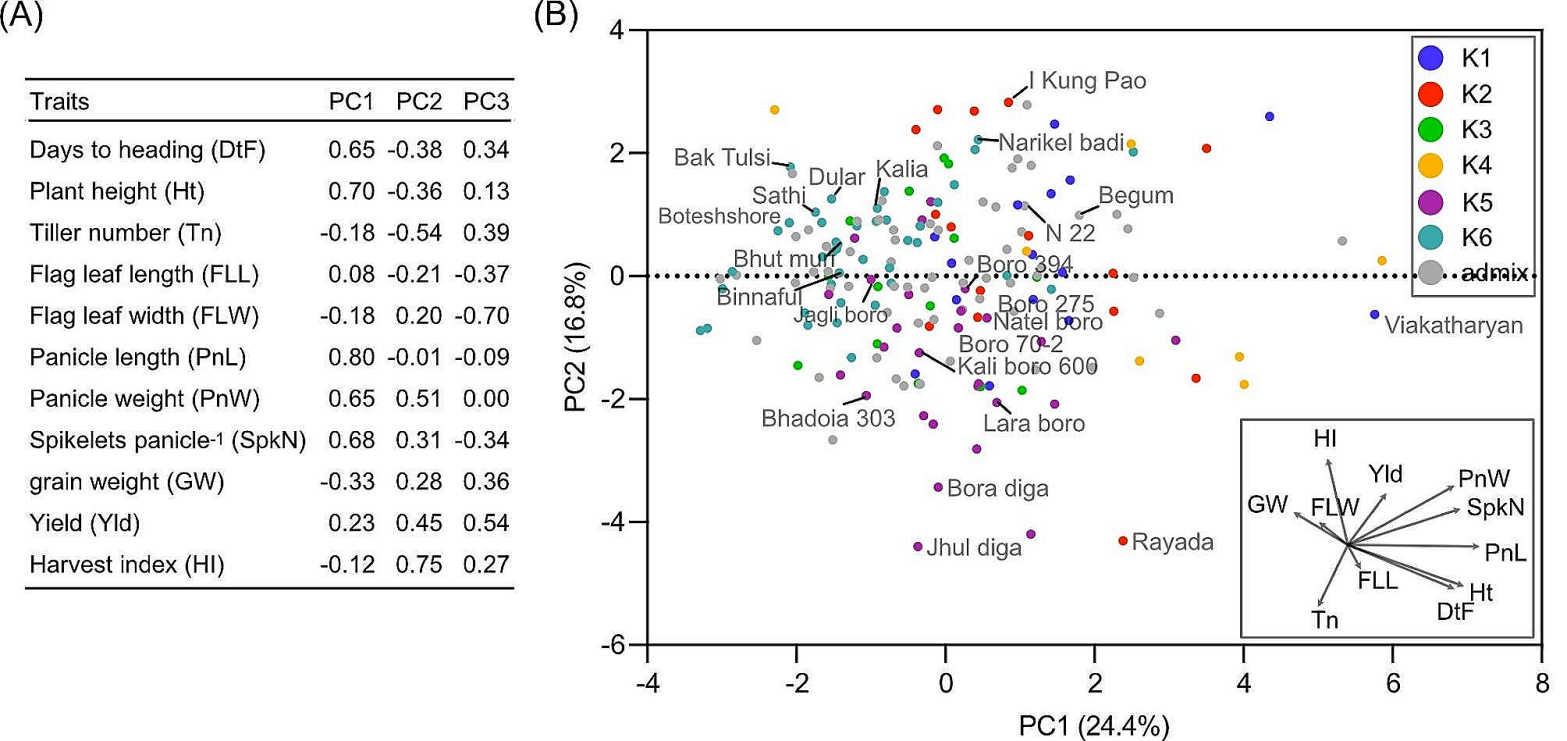
**Principal component analysis for morpho-agronomic traits in 181*aus*cultivars.A** Summary of first three PCs for 11 traits. **B** PCA biplot showing the distribution of *aus* accessions based on the trait loadings (shown in the inset) on the first two PCs. The accessions were colour coded according to their classification into six genetic subgroups based on 399 K SNP dataset

We performed a PCA using 11 quantitative agronomic traits to investigate the relationships among traits and the factors underlying the trait variation. Altogether, three principal components, PC1, PC2 and PC3, were selected which explained 24.4%, 16.8% and 13.9% of the trait variance, respectively (Fig. 4A). The PC1 explained variation in agro-morphological traits arising from plant architecture and flowering as it was positively loaded with days to flowering, plant height, panicle length, panicle weight and spikelets per panicle. This result suggested that accessions with high PC1 scores exhibited larger and heavier panicles, taller plants and longer days to heading. The grain weight and tiller number loaded negatively on PC1, indicating a trade-off relationship between grains per panicle and 1000-grain weight, as well as between tiller number and panicle size. Similarly, PC2 mostly explained variation in traits directly related to grain yield by showing positive loading by panicle weight, grain yield and harvest index. On PC2 days to heading and tiller number showed negative loading on PC2, suggesting a negative correlation between days to flowering and harvest index which is consistent with the observation that prolonged vegetative growth due to late heading leads to a reduced harvest index. The PC3 also explained variation for grain yield. The accessions with high PC3 scores mostly exhibited higher grain yield.

The correspondence between the patterns of genetic and morphological diversity was checked by performing a biplot analysis of PC1 and PC2 (Fig. 4B). The boro and deep-water accessions (belonging to K6) were found to be morphologically distinct from the drought tolerant cultivars of K5. However, the genetic distance between these two groups is less (*F*_*ST*_ = 0.183). The rayada cultivar remained distinct from the rest of the *aus* both genetically and agronomically. The genetic distinctness of cultivars of K4 was also reflected in their morphological clustering. These results indicated that the selection of diverse stress tolerant and high yielding *aus* cultivars is feasible for breeding programmes.

### GWAS for PC Scores

We conducted GWAS using the first three PCs (PC1 to PC3) as well as 11 traits using a 458 K SNP set to identify the key loci controlling agronomic characteristics of *aus* rice. The normality of the PCs and individual traits was checked using the Kolmogorov-Smirnov test. PC1 showed slight deviation from normality (*P* = 0.0117), while PC2 and PC3 showed normal distribution. Among the 11 morpho-agronomic traits, except for 1000-grain weight and yield plot^− 1^, we found significant deviations from the normal distribution. This result corroborates with earlier observations (Yano et al. 2019) that PCA can transform skewed data to a normal distribution, which is useful to improving the statistical power of GWAS.

**Fig. 5:**
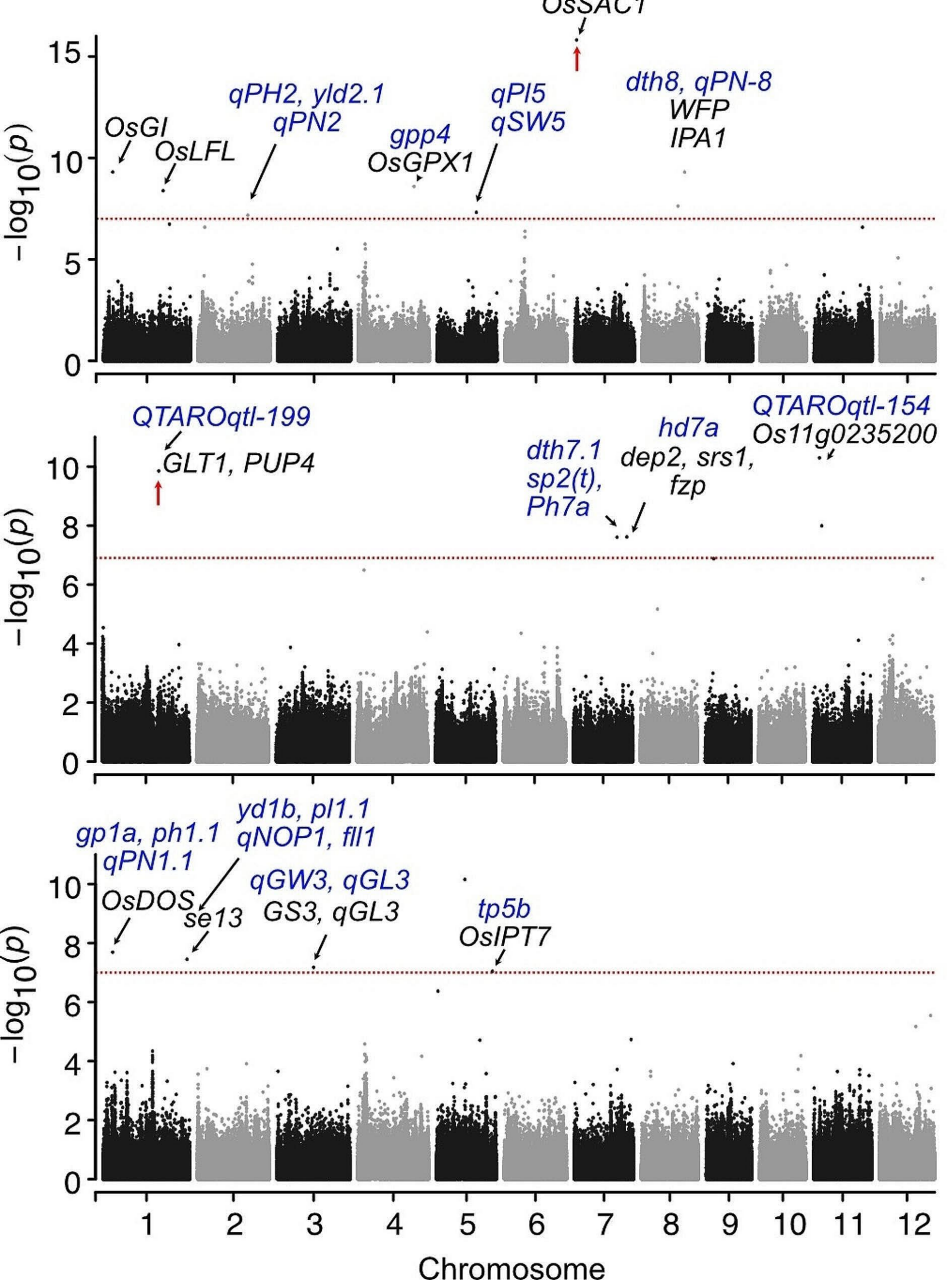
**GWAS for morpho-agronomic traits in*aus*rice.** Manhattan plots of PC1, PC2 and PC3 are shown. Horizontal red dotted lines represent the significant threshold for the study. The colocalization of previously identified QTLs (blue font) and genes (black font) are indicated by black arrows. The red arrows indicate the peaks we studied further

Except for PCs, grain weight and yield, we used log_10_-transformed values of the rest of the traits to conduct GWAS using the FarmCPU model with corrections for kinship bias (Fig. 5; Fig. S6-S7). GWAS from PCs identified 18 peaks (PC1 = 8, PC2 = 5 and PC3 = 5) with a -log_10_(*P*)- value that exceeded the Bonferroni cut-off. The significant associations detected for all three PCs are listed together along with their phenotypic variance (Table 1). Interestingly, some of the peaks detected for different PCs on Chr1 (Chromosome1), Chr5, Chr5, Chr8 and Chr11 were found to be coinciding, indicating that these peaks may represent a common flanking region (Table 1). We defined the QTL regions corresponding to the significant SNPs by expanding the upstream and downstream flanking regions according to the chromosome-wide LD decay distance analyzed in this study (Fig. S8).

**Table 1:** QTLs for morpho-agronomic traits identified in GWAS using principal components in 181 *aus* rice germplasm

Trait	QTL	Chr	Flanking region (Mb)	Peak SNP position	Ref allele	-Log_10_(P)	MAF	Effect	PVE
PC1 (DtF, Ht, PnL, PnW, SpkN)	*qPC1-1.1*	1	4.36–4.68	4,521,776	G	9.30	0.10	-0.69	5.06
	*qPC1-1.2*	1	29.49–29.81	29,651,634	C	8.38	0.21	0.53	1.47
	*qPC1-2.1*	2	24.78–24.90	24,835,927	G	7.18	0.30	-0.38	2.05
	*qPC1-4.1*	4	27.76–27.85	27,801,414	G	8.59	0.20	0.37	2.05
	*qPC1-5.1*	5	19.42–19.81	19,616,683	G	7.32	0.23	0.42	5.80
	*qPC1-7.1*	7	0.73–1.05	888,054	A	15.81	0.16	-1.04	7.97
	*qPC-8.1*	8	17.81–18.19	18,001,305	A	7.63	0.16	-0.56	5.13
	*qPC1-8.2*	8	21.12–21.51	21,316,516	T	9.30	0.13	0.76	1.11
PC2 (PnW, Yld, HI)	*qPC2-1.1*	1	27.93–28.25	28,087,887	T	9.85	0.15	0.44	10.53
	*qPC2-7.1*	7	21.54–21.86	21,700,005	T	7.61	0.27	0.33	2.58
	*qPC2-7.2*	7	26.45–26.77	26,611,742	C	7.62	0.12	0.50	1.23
	*qPC2-11.1*	11	3.35–3.48	3,412,660	G	10.30	0.48	-0.34	3.98
	*qPC2-11.2*	11	4.49–4.62	4,552,359	T	7.99	0.15	-0.54	1.60
PC3 (Tn, GW, Yld)	*qPC3-1.1*	1	4.62–4.94	4,789,451	G	7.69	0.07	0.46	6.20
	*qPC3-1.2*	1	41.66–41.98	41,821,709	A	7.45	0.33	0.39	6.51
	*qPC3-3.1*	3	17.99–18.45	18,218,891	C	7.18	0.06	-0.55	2.07
	*qPC3-5.1*	5	13.96–14.35	14,156,143	G	10.16	0.17	0.48	5.86
	*qPC3-5.2*	5	27.71–28.10	27,904,519	C	7.04	0.35	0.26	0.37

DtF, Days to 50% flowering; Ht, Plant height, PnL, Panicle length, PnW, Panicle weight, SpkN, Spikelets per panicle; Yld, Yield; HI, Harvest index; Tn, Tiller number, GW, 1000-grain weight; Chr, Chromosome; MAF, Minor allele frequency; PVE, Phenotypic variation explained %.

DtF, Days to 50% flowering; Ht, Plant height, PnL, Panicle length, PnW, Panicle weight, SpkN, Spikelets per panicle; Yld, Yield; HI, Harvest index; Tn, Tiller number, GW, 1000-grain weight; Chr, Chromosome; MAF, Minor allele frequency; PVE, Phenotypic variation explained %.

The PCA indicated that PC1 is representative of plant architecture and flowering, while both PC2 and PC3 are representative of grain yield. In this study, we focussed on the genetic loci responsible for plant architecture or grain yield as a trait rather than focusing on specific traits. Therefore, we focussed on the results of GWAS using PCs. The significant associations identified for each PC were colocalized with several previously reported QTLs for yield and agronomic traits when searched in databases (Fig. 5). Notably, the significant marker-trait associations (MTAs) identified in the current study were overlapping with many cloned genes such as, for PC1, *OsGI* (*GIGANTEA*) regulating days to heading, *OsGPX1* (*Plant Glutathione peroxidases 1*) influencing plant height, spikelet number and root development, *OsMADS15* influencing flowering time and plant architecture, and *WFP*/ *IPA1* (*WEALTHY FARMERS PANICLE/ IDEAL PLANT ARCHITECTURE 1*) controlling yield and plant architecture. For PC2, important genes coincided with the index SNPs were *OsGLT1* (NADH-glutamate synthase 1) controlling yield, *dep2/SRS1* (*DENSE AND ERECT PANICLE 2*) regulating panicle size, *fzp* (*frizzy panicle*) influencing panicle and yield traits, and *SP1* (*Os11g0235200*, *SHORT PANICLE 1*) for panicle traits.

Important genes identified for PC3 were: *OsDOS* (*DELAY OF SENESCENCE*) regulating crop maturity, *SE13* (*PHOTOSENSITIVITY 13*) controlling heading date and yield, *GS3* and *qGL3* for grain traits, and *OsIPT7* (*Adenosine phosphate isopentenyltransferase 7*) influencing yield traits. Furthermore, on Chr4 prominent peaks were detected at position ~ 3.540 Mb for both PC1 and PC3. This association corresponds to the QTL *qSNP-4a* and *spp4-2*, both reported for spikelets panicle^− 1^. On Chr6 another prominent peak was observed for PC1 at position ~ 10.116 Mb which has been identified as a QTL hotspot region harbouring QTLs like *qSNP6* and *gp6* (spikelet number), *gw6* (1000-grain weight), *qTN2-6-1* (tiller number at maturity), *qPH2-6-1* and *Ph6* (plant height at maturity), *qGY*_*6.1*_ (grain yield), and *qHD6-1* (heading date). An important gene present in this region is *Hd1* responsible for regulating photoperiodic flowering in rice. Furthermore, we observed that none of the GWAS signals identified for PCs were identified when performing GWAS using individual traits except for *qPC1-2.1*, which is a hit for spikelet number.

### Phenotypic Effect of the Allelic Variations of MTAs

Among the eight QTLs identified for PC1, significant differences in PC scores were observed between the accession groups with the reference (‘ref’) and alternate allele for all QTLs except for *qPC1-1.2* and *qPC1-1.2*, possibly due to their low MAF and smaller effect size, respectively (Fig. S9; Table 1). The loci identified for PC1 have shown significant allelic differences for days to heading and many of the plant architectural traits supporting our assumption for PC1 (Fig. S9). However, QTLs like *qPC1-1.1, qPC1-5.1, qPC1-8.1* and *qPC1-8.1* also explained the variation for grain yield. Except for *qPC2-11.2*, the rest of the QTLs identified for PC2 showed significant differences in PC2 scores (Fig. S10). All the PC2 QTL have explained variation for grain yield traits as expected from the trait loadings on PC2. Whereas, some of these QTL have also explained variation for flowering and architectural traits. We found significant differences in PC3 scores for two QTLs – *qPC3-1.2*, and *qPC3-5.1*. Both these QTL have explained variation for yield traits as we all as days to heading and spikelet number. Although the rest of the PC3 QTLs did not show significant variation in PC scores, but these QTLs have influenced 1000-grain weight and yield. Overall, these results indicated that the GWAS using PCs could identify QTLs for the traits which showed lesser loadings on a particular PC.

### Haplotype Analysis of Potential QTLs

**Fig. 6:**
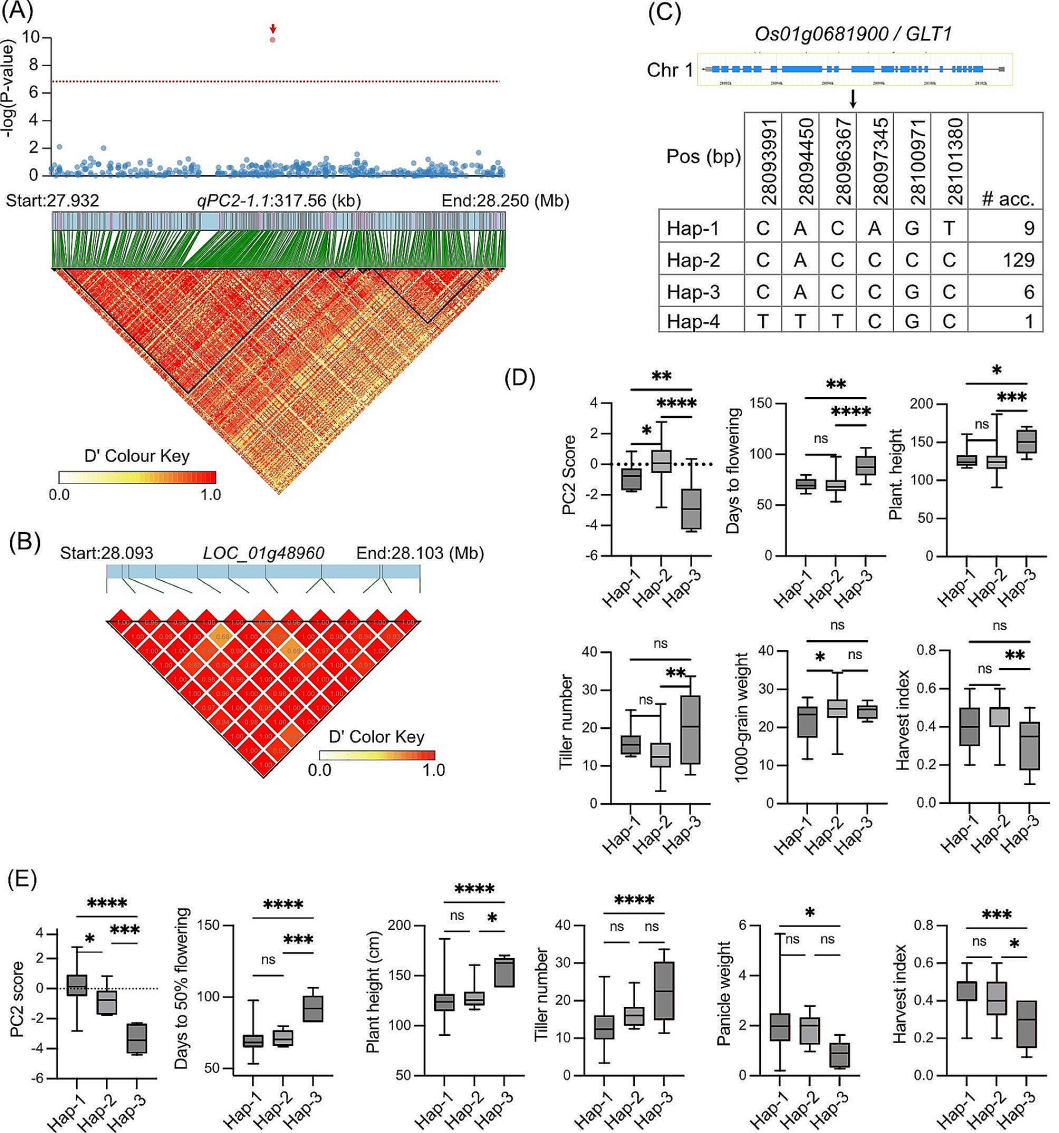
**Haplotype analysis within*qPC2-1.1***. **A** Local Manhattan plot and LD heat map for *qPC2-1.1* on Chr 1. The red arrow (Top panel) indicates the position of *OsGLT1.***B** LD heat map of *LOC_Os01g48960*. **C** Structure and DNA polymorphism of *OsGLT1*. **D** Box plots of PC2 score and agro-morphological traits for three haplotypes Hap-1 (*n* = 9), Hap-2 (*n* = 126) and Hap-3 (*n* = 6) of *OsGLT1*. **E** Box plots of PC2 score and agro-morphological traits for three haplotypes Hap-1 (*n* = 116), Hap-2 (*n* = 11) and Hap-3 (*n* = 7) of *OsPUP4*. Box edges represent the 0.25 and 0.75 quantiles, with the median values shown within boxes. Whiskers extend to the most extreme point, which is no more than 1.5 times the interquartile range. Differences between the haplotypes were statistically tested using multiple comparisons with Tukey’s t test (ns, not significant, *, **, ***, and **** represent *P* value < 0.05, < 0.01, < 0.001, and < 0.0001, respectively)

The *qPC2-1.1* (peak SNP Chr1: 28,087,887) explained the highest variance for PC2 which represents grain yield, panicle weight and harvest index. We examined this QTL for genes that could influence grain yield and component traits. The QTL region was delineated to 27.93–28.25 Mb and contained 374 SNPs (Fig. 6A). In total, 58 genes including two retrotransposons are present in *qPC2-1.1* (www.rapdb.dna.affrc.go.jp; Dataset S5). Among these genes, *Os01g0681900*, located 3,321 bp downstream of the peak SNP, and *Os01g0680200*, located 90.3 kb upstream of the peak SNP, were reported to be associated with grain yield and other yield-related traits.

*Os01g0681900* (synonymous *OsNADH-GOGAT1* or *GLT1*), annotated as glutamate synthase or NADH-DEPENDENT GULTAMATE SYNTHASE 1, influences grain yield by affecting panicle number, tiller number, tillering ability. It also regulates nitrogen-carbon metabolomes (Yang et al. 2016), and plays a key role in the transcriptional regulation of ammonium-responsive genes (Kojima et al. 2023). The LD plot based on 12 SNPs within *GLT1* indicated strong linkage among the SNPs (Fig. 6B). We identified four haplotypes (named Hap-1 to Hap-4) using the six non-synonymous SNPs in the current 181 *aus* panel (Fig. 6C). Hap-2 was most frequent (present in 72% of the accessions), while Hap-4 was detected in a single accession. The average PC2 score for the accessions carrying Hap-2 was significantly higher than those with either Hap-1 or Hap-3 (Fig. 6D), indicating that *GLT1* is associated with PC2. The haplotypes exhibited significant differences for most of the agro-morphological traits except for panicle weight and yield (*P* = 0.051) (Fig. 6D). The Hap-3 accessions showed significantly longer days to heading as well as taller plants than both Hap-1 and Hap-2 accessions (Fig. 6D). Although there were non-significant differences in grain yield among the haplotypes, Hap-2 showed considerably higher level of yield and recorded the highest 1000-grain weight and harvest index among the haplotypes. Interestingly, accessions with Hap-3 showed the highest tiller number but the lowest harvest index, indicating that those may had either low spikelet fertility or had produced many non-productive tillers. In the *aus* panel, Hap-1 was predominant in accessions from India, Bangladesh and Sri Lanka. While, Hap-3 accessions are mostly confined in Bangladesh and adjoining Assam. Grouping two ‘diga’ (known to be of deep-water ecology) cultivars in Hap-3 suggested that this haplotype is primarily unique to deep-water accessions. The average elongation ability under submergence stress of the six accessions belonging to Hap-3 was 149.3% (data from a separate study).

The other gene, *Os01g0680200 / OsPUP4* (*PURINE PERMEASE 4* syn. *Big Grain 3*), in *qPC2-1.1* is reported to regulate grain size, along with several other traits like grain number, secondary branch number, tiller angle, days to heading, 1000-grain weight, and plant height. *PUP4* was suggested to be involved in the long distance transport of cytokinin, by reinforcing cytokinin loading into vascular bundle cells (Xiao et al. 2019) (Dataset S5). We identified three haplotypes (Hap-1 to Hap-3) of this gene in the current *aus* genotypes based on three non-synonymous SNPs. Hap-1 was present in 90.7% while, Hap-3 was found in 3.5% of the *aus* panel. The haplotypes showed significant differences for PC2 scores along with days to heading, plant height, tiller number, panicle length and harvest index (Fig. 6E). Accessions possessing Hap-1 of *PUP4* were early to flower, shorter in height, had lesser tillers with heavier panicles, and higher harvest index. Notably, the deep-water accessions having Hap-3 of *PUP4* also carried the Hap-3 of *GLT1*. These accessions were considerably late maturing and taller with lightweight panicles.

**Fig. 7:**
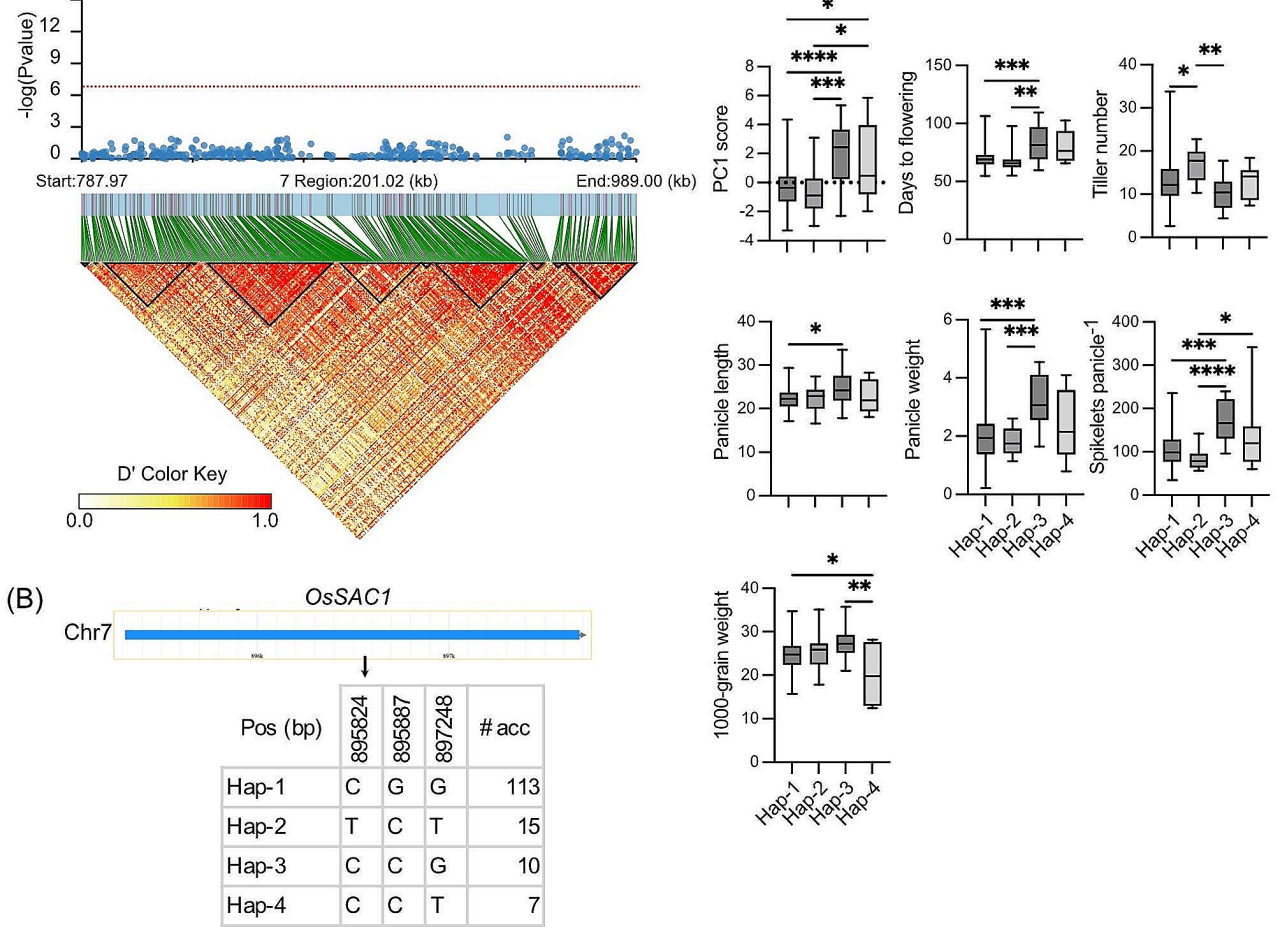
**Haplotype analysis within*qPC1-7.1***. **A** Local Manhattan plot and LD heat map for *qPC1-7.1* on Chr 7. The red arrow (Top panel) indicates the position of *LOC_Os07g02520/ Os07g0116300.***B** Structure and polymorphism of *OsSAC1*. Four haplotypes were detected in 147 *aus* accessions. In the rest of the accessions either of the SNPs were missing or in heterozygous condition. (C) Box plots show significant variation among the haplotypes for PC1 score and agronomic traits. Box edges represent the 0.25 and 0.75 quantiles, with the median values shown within boxes. Whiskers extend to the most extreme point, which is no more than 1.5 times the interquartile range. Differences between the haplotypes were statistically tested using Tukey’s test (*, **, ***, and **** represent *P* value < 0.05, < 0.01, < 0.001, and < 0.0001, respectively)

On Chr7 the *qPC1-7.1* also explained high phenotypic variance and had a high effect size for PC1 which represents days to flowering and plant architectural traits. This QTL was delineated to 0.73–1.05 Mb and contained 48 genes (Dataset S6; https://rapdb.dna.affrc.go.jp/). Among the genes, *Os07g0116300*, annotated as *OsSAC1* or *SUGAR ACCUMULATION 1*, is previously reported to influence grain yield, spikelet number, plant height, panicle length, 1000-grain weight, starch content, and photosynthetic rate (Zhu et al. 2017). The *qPC1-7.1*, identified in the present study, is a novel one as no QTLs for yield or plant architectural traits were earlier reported in this region (https://snp-seek.irri.org/), except *qSS-7a* for hybrid sterility (Li et al. 2008). LD block analysis of *qPC1-7.1* based on 342 SNPs revealed 5 large and 6 small blocks (Fig. 7A). For *SAC1*, we identified six haplotypes using three non-synonymous SNPs within the gene. Out of these, each of the haplotypes 5 and 6 were detected in a single accession. Hence, we presented the results of haplotypes 1 to 4 (Hap-1 to Hap-4) (Fig. 7B). Hap-1 was the most frequent, detected in 77% of the accessions. While, Hap-4 was the least frequent and observed in 5% of the accessions. The accessions belonging to Hap-3 had the highest average PC1 score, and thereby, also had the highest values for days to flowering, panicle length, panicle weight, spikelets panicle^− 1^ (Fig. 7C). This haplotype also recorded the highest average 1000-grain weight. The accessions with *OsSAC1* Hap4 showed wide variation for PC1 score and other traits like panicle length and 1000-grain weight. The accessions with Hap-1 and Hap-2 were early maturing but recorded lower values for panicle weight and spikelets panicle^− 1^. We found that *OsSAC1* Hap-3 was prevalent in the accessions belonging to the genetic cluster K2 and K4. It has been shown that the K2 cluster, which also include the cultivar rayada, is characterized by heavier panicles with higher spikelet number. As well as both K2 and K4 mostly included longer duration accessions with longer and heavier panicles, and higher spikelets panicle^− 1^. The Hap-3 has been found to be geographically most widespread while Hap-2 is mostly confined in Bangladesh. Within the *qPC1-7.1* another gene *Os07g0119000* (annotated as *OsMAPKKK11, MAPK Kinase Kinase 11*) seems to have a role in rice growth and development (Duan et al. 2014; Guo et al. 2018, 2020), in addition to coordinating resistance to biotic and abiotic stress responses(Yamada et al. 2017; Chen et al. 2021) (Dataset S6).

**Fig. 8:**
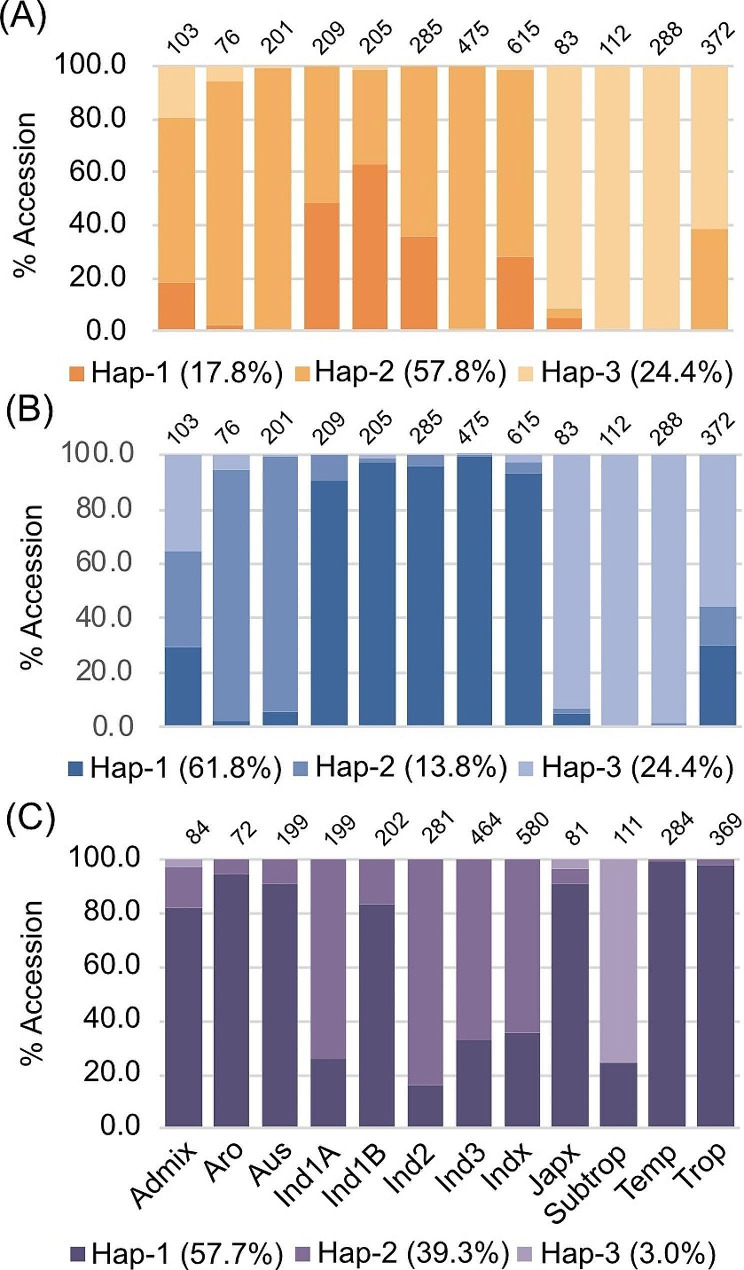
**Gene haplotype distribution in 3,000 rice genome (3 K-RG) panel.** (A) frequency of three haplotypes of *GLT1* in different rice subgroups. (B) frequency of three haplotypes of *PUP4* in different rice subgroups. (C) frequency of three haplotypes of *SAC1* on Chromosome 7 in different rice subgroups. The frequency of each haplotype in 3 K-RG panel is mentioned within the parentheses. Haplotype analysis was performed using the non-synonymous SNPs within the genes using the Rice SNP-Seek Database (https://snp-seek.irri.org/_snp.zul). Grouping of rice germplasm into 12 subgroups was done following Wang et al. (2018). Admix, admixture; Aro, aromatic; Aus, aus; Ind1A, indica 1 A; Ind 1B, indica 1B, Ind2, indica 2; Ind3, indica 3; Indx, indica admixture; Japx, japonica admixture; Subtrop, subtropical japonica; Temp, temperate japonica; Trop, tropical japonica. Number of accessions in each subpopulation is mentioned on the top of each bar

We further compared the haplotype frequency of *GLT1, PUP4* and *SAC1* among the 3,002 accessions from 3 K-RG panel using Rice SNP-seek web-based applications. Three haplotypes were detected for *GLT1* based on 11 SNPs (out of which four appeared in our *aus* panel). The haplotypes showed differential distribution among the *Oryza sativa* subpopulations: Hap-1: *indica*; Hap-2: *indica* subgroups; *aus* + *indica* + *aro* + *tropical japonica*; and Hap-3: *japonica* subgroups (Fig. 8A). Similarly, three haplotypes were also detected for *PUP4.* The *aus* and *aromatic* subpopulations carry separate gene haplotypes than both *indica* and *japonica* subpopulations (Fig. 8B). For *SAC1*, out of three haplotypes, *aus* subpopulation mostly carries similar haplotypes found in *aromatic, temperate japonica, tropical japonica*, and *indica 1B* subpopulations (Fig. 8C).

## Discussion

Understanding the genetic structure and origins of morphological and developmental variations in *O. sativa* is vital for bolstering global food security. Extensive investigations on rice population structure have consistently identified two major varietal groups, denoted as sub-species: *Indica* and *Japonica* (Wang et al. 2018). This classification has ancient Chinese roots, known as *Hisen*/*Sen* and *Keng*/*Geng*, and was subsequently substantiated by morphological and serological distinctions, along with the presence of partial reproductive barriers (Kato 1928; Morishima and Oka 1981). The *Japonica* varietal group was further divided into *tropical japonica* and *temperate japonica* (Oka 1958). Additionally, the *Javanica* group emerged based on gross morphological distinctions and geographical distribution (Morishima and Oka 1960). Apart from these three primary groups, various minor varietal groups, such as aus, ashina, boro, rayada, basmati, and sadri, are cultivated across the Indian subcontinent. While these groups may lack significant morphological disparities compared to *Indica* and *Japonica*, recent studies employing isozyme loci and molecular marker systems have established some as distinct genetic groups within *O. sativa* (Glaszmann 1987; Garris et al. 2005; Wang et al. 2018).

Glaszmann’s (1987) comprehensive work categorized 1,688 *O. sativa* accessions into six varietal groups, encompassing two major (*Indica* and *Japonica*), two minor (*aus* and *aromatic*), and two satellite (*ashina*/deep-water and *rayada*). Subsequent studies, such as Garris et al. (2005), proposed a widely accepted classification consisting of *indica, aus, aromatic, tropical japonica*, and *temperate japonica*. However, these studies omitted many accessions from the satellite groups originating in Bangladesh and northeastern India (Group III and Group IV of Glaszmann 1987). Research into the population structure of Asian rice is often constrained by the limited representation of rice cultivars from specific ecological regions, which may obscure finer population structures. For instance, while the analysis of the 3 K-RG panel, comprising millions of SNPs, offered an enhanced resolution of within-species diversity, it failed to fully unravel the structure within the circum-*aus* group, encompassing aus, boro, ashina, and rayada types (Wang et al. 2018). However, another study incorporating various Chinese accessions successfully elucidated the structure within the circum-*aus* group (Wang et al. 2014).

In this study, we specifically focussed on comprehending the finer population structures within the circum-*aus* group by scrutinizing the aus/boro accessions from the 3 K-RGP dataset, an area where our understanding of population genomic diversity is quite limited. Our analysis unveiled the existence of six sub-groups, supporting the differentiation of previously identified *aus* subgroups, encompassing aus, ashina, and rayada types. Significantly, these sub-groups displayed distinct geographic patterns. In prior research by Travis et al. (2015), the genetic structuring of 345 *aus* cultivars, predominantly originating from Bangladesh, Assam, and eastern India, was examined using 384 SNPs. Travis et al. identified two distinct groups within the *aus* varietal category. Geographically, this group is distributed across south and west Asia, extending from Iran to Assam along the Himalayas (Glaszmann 1987), with its center of diversity situated in Bangladesh and the eastern to northeastern regions of India (Civáň et al. 2015). These rice varieties are cultivated under a wide range of hydric conditions, from irrigated regions in Pakistan to drought-prone uplands in Bangladesh and eastern India, as well as deep-water environments in Bangladesh and northeastern India. Consequently, they have developed numerous adaptive traits to thrive in these diverse environments, leading to genetic variations at the DNA level and intricate fine-scale population structures.

Our findings revealed that when assessing genetic differentiation between aus and boro ecotypes at *K* = 2, they appeared to be genetically close, sharing a cluster. However, at *K* = 6, boro cultivars exhibited some degree of differentiation, primarily forming a cluster under K5. This suggests that while there is genetic proximity between aus and boro ecotypes, finer genetic distinctions become apparent when examining a more detailed population structure. At both levels of structuring, rayada remained at a separate cluster, aligning with previous research findings (Wang et al. 2014; Travis et al. 2015). Interestingly, our study found that many deep-water cultivars, categorized as ashina, exhibited a stronger genetic affinity with boro cultivars, a phenomenon not previously reported. Furthermore, we observed that the majority of *aus* accessions originating from countries outside the Indian subcontinent tended to cluster separately, as illustrated in Fig. 1. Pairwise genetic distance calculations among the genetic clusters (K1-K6) revealed that drought-tolerant *aus* accessions (belonging to K3) may have dispersed further to regions like Sri Lanka and Pakistan, likely due to their stress tolerance. Similarly, rayada cultivars may have also extended beyond Bangladesh to central India. However we could not find a possible reason the spread of rayada types. Overall, there was a connection between the geographical distribution of *aus* sub-groups and agro-ecological diversity in Southern Asia. After comparing the distribution of the accessions along the five agro-ecological zones (FAO’s global agro-ecological zones modified by Gumma et al. 2022), we found that K1 is distributed in humid tropics (Zone 5). K2 and K3 are distributed along the Zones- 1, 2 and 3 representing arid tropics, semi-arid subtropics and semi-arid tropics, respectively. K6 is widespread in sub-humid tropics (Zone 4), while K5 is distributed along the transition zone of Zones- 4 and 5. Only the accessions of K4 are not confined to any agro-ecological zones.

When constructing phylogenetic trees from genome-wide data, it became evident that *aus* cultivars cluster within the *Indica* clade, indicating their greater genetic similarity with *Indica* rice. This genetic closeness aligns well with their morphological similarities. However, several studies have also suggested a distinct origin for *indica* and *aus* varietal groups (Schatz et al. 2014; Civáň et al. 2015). The *aus* cultivars form a distinct cluster from both *indica* and *japonica* when neighbor-joining trees are constructed from the ‘domestication sweep’ regions (Civáň et al. 2015), highlighting the potential for a separate origin of *aus*.

Based on this study and previous evidence, it appears that the circum-*aus* group represents an evolutionary development of *aus* rice. Further genome-wide surveys with increased sample sizes of *aus, indica*, and wild species from the Indian subcontinent could provide greater clarity on the origin of the *aus* group. Moreover, the wide agro-morphological variations observed within this group enhance the potential for utilizing *aus* accessions in rice breeding programs to create novel genetic variations for yield-related traits.

In our study, we employed high-density marker data to unravel the intricate genetics governing yield traits in *aus* rice. This was achieved through mixed-model GWAS analyses using principal component (PC) scores derived from 11 agro-morphological traits. PCA, a dependable approach for extracting the underlying variability from numerous correlated traits, was utilized to create PC scores, which can be considered as composite variables. The application of GWAS with PC scores has proven effective in reducing Type I error rates by circumventing multiple testing, a method employed in both human and plant systems (He et al. 2008). Additionally, PCA normalizes skewed individual trait data, improving the reliability of GWAS results (Goh and Yap 2009). Notably, in rice, GWAS using PC scores has demonstrated higher power in detecting loci that might be overlooked when using individual traits as dependents (Yano et al. 2019).

Our PCA analysis of 11 agro-morphological traits unveiled that PC1 explained 24% of the variation in flowering and plant architectural traits, while PC2 captured 17% of the variations in grain yield traits, such as panicle weight, yield, and harvest index. PC3 captured 14% of the variation for grain yield. Utilizing PC scores for GWAS proved to be more effective in identifying significant associations for yield-related traits compared to using individual traits for GWAS. Moreover, several peak SNPs coincided with previously reported QTLs and genes, strengthening the reliability of GWAS results. For instance, the genes associated with PC1 score were linked, either falling within or being in LD with *OsGI, OsGPX1, OsMADS15*, and *IPA1*, which regulate days to flowering, plant height, spikelet number, root development, panicle architecture, and grain yield. SNPs identified for PC2 were linked to genes such as *OsGLT1, dep2, fzp*, and *SP1*, while PC3 results revealed associations with several genes influencing grain size and yield, including *OsDOS, SE13, GS3, GL3*, and *OsIPT7*.

Further exploration of the identified QTLs (*qPC2-1.1* and *qPC1-7.1*), explaining higher phenotypic variance with larger effect sizes, uncovered potential candidate genes for agro-morphological traits in *aus* rice germplasm. QTL *qPC2-1.1*, located on Chromosome 1, significantly explained variation for PC2, representing grain yield, panicle weight, and harvest index. Examination of gene models suggested that *OsNADH-GOGAT1* (*GLT1*) and *OsPUP4* are the likely candidate genes for this QTL. *GLT1* influences various yield-related traits, and nitrogen-carbon metabolism by regulating ammonium-responsive genes (Funayama et al. 2013), and is expressed in roots, young leaves, and grains. Rice *GLT1* mutants exhibit reduced tillering (Tamula et al., 2010). In *aus* rice, we detected four haplotypes of *GLT1*, with one particular haplotype (Hap-2) present in 72% of the accessions. This haplotype was associated with early maturation, higher 1000-grain weight, and increased harvest index.

Our analysis of the 3 K-RGP showed that *aus* rice carries a specific *GLT1* haplotype, prevalent in indica (Ind3) accessions from southeast Asia, as well as in aromatic and tropical japonica rice. Haplotypes of *GLT1* exhibit significant differentiation between rice varietal groups (Yang et al. 2016). Another potential gene within *qPC2-1.1* is *Big Grain 3*/*OsPUP4*, which influences various agro-morphological traits. The *PUP4* gene family includes 12 members involved in cytokinin transportation. Activating *OsPUP4* results in increased grain size (Xiao et al. 2019). *OsPUP7*, a homolog of *OsPUP4*, regulates multiple phenotypic traits in rice, including plant height, grain size, and days to heading. Haplotypes of *PUP4* in *aus* accessions differed for many traits, suggesting this gene is another candidate for *qPC2-1.1*. The distribution of *PUP4* haplotypes also varies among *indica, aus*, and *japonica*.

The candidate gene for *qPC1-7.1* appears to be *OsSAC1*, involved in regulating sugar partitioning in carbon-demanding juvenile leaves and leaf sheaths. Although the function of *OsSAC1* is not fully characterized, this gene likely contributes to building the carbon skeleton in rice plants. Previously, no QTLs for flowering or plant architectural traits were reported in the *qPC1-7.1* region, highlighting the effectiveness of GWAS using PC scores in identifying QTLs not detectable through individual traits. Haplotype frequencies of *OsSAC1* vary between *indica* and *japonica*, with the dominant gene haplotype in *aus* rice being the most frequent in *temperate*- and *tropical-japonica*, along with modern *indica* varieties. Overall, from the distribution of haplotype frequencies of *OsGLT1, OsPUP4*, and *OsSAC1*, it is evident that *aus* rice possesses different gene haplotypes compared to most *indica* rice. These findings support the recent hypothesis of *O. sativa* evolution, suggesting separate origins of *indica, aus*, and *japonica* in different geographic regions, with *aromatic* rice likely originating from hybridization between *aus* and *japonica* (Civáň et al. 2015; Civáň et al. 2019). It would be interesting to study whether the haplotypes we have identified in *aus* also has the same effects in other varietal groups, or whether they are modified by these differing genetic backgrounds; examining this issue may help in providing new breeding material for a wider range of rice populations.

## Conclusions

- Our investigation focused specifically on unravelling the finer population structures within the circum-*aus* varietal group. We unveiled the existence of six *aus* sub-groups, and highlighted their distinct geographic patterns. Our findings emphasized the genetic proximity between aus and boro ecotypes, while revealing the genetic distinctness of rayada cultivars. Notably, many deep-water cultivars (categorized as ashina), displayed a strong genetic affinity with boro cultivars. Furthermore, our research revealed that *aus* accessions from countries outside the Indian subcontinent tend to cluster separately, indicating genetic distinctions.
- GWAS analyses with PC scores derived from 11 agro-morphological traits proved more effective in identifying significant associations for yield-related traits compared to using individual traits in GWAS. Our investigations unveiled the potential candidate genes for QTLs, such as *OsNADH-GOGAT1* (*GLT1*), *OsPUP4*, and *OsSAC1*, offering valuable insights into the genetic basis of grain yield and other agronomical traits.
- In conclusion, this research contributes to a deeper understanding of the genetic intricacies of *aus* rice and offers insights into its evolutionary history. These findings are important for future rice breeding programs aimed at improving yield-related traits and enhancing global food security.

### Electronic Supplementary Material

Below is the link to the electronic supplementary material.

Supplementary Material 1

Supplementary Material 2

## Data Availability

All data generated or analysed during this study are included in this published article and its supplementary information files.

## Abbreviations

GWAS: Genome-wide association study
PCA: Principal component analysis
PC: Principal component
3 K-RGP: 3,000 Rice Genome Project
BAAP: Bengal Assam Aus Panel
LD: Linkage disequilibrium
Chr: Chromosome
MAF: Minor allele frequency
DtF: Days to 50% flowering
Ht: Plant height
PnL: Panicle length
PnW: Panicle weight
SpkN: Spikelets per panicle
Yld: Yield
HI: Harvest index
Tn: Tiller number
GW: 1000-grain weigh
PVE: Phenotypic variation explained
MTA: Marker-trait association
QTL: Quantitative trait locus
