# Supplementary material for: Exploring Genetic Diversity within aus Rice Germplasm: Insights into the Variations in Agro-morphological Traits
Source: Rice (N Y). 2024 Mar 25;17:20. doi: 10.1186/s12284-024-00700-4 (PMC10963711; doi:10.1186/s12284-024-00700-4)
Supplement: Supplementary file 2 — Supplementary Material 2 [file 12284_2024_700_MOESM2_ESM.docx]

***Supporting information***

**Article title:** Exploring genetic diversity within aus rice germplasm: insights into the variations in agro-morphological traits

**Authors:** Puranjoy Sar, Sonal Gupta, Motilal Behera, Koushik Chakraborty, Umakanta Nongkham, Bibhash Chandra Verma, Amrita Banerjee, Prashantkumar S. Hanjagi, Debarati Bhaduri, Sandip Shil, Jitendra Kumar, Nimai Prasad Mandal, Paresh Chandra Koley, Michael D. Purugganan, Somnath Roy

*Following supporting information is available for this article*

**Fig S1.** Plots of cross validation error values generated from ADMIXTURE software for (A) over all 181 *aus* accessions, (B) Bangladesh *aus* accessions and (C) Indian *aus* accessions.

**Table S1.** *F_st_* divergences between estimated *aus* subpopulations (*K* = 1-6) detected through ADMIXTURE analysis.

**Table S2.** Descriptive statistics of quantitative traits in the studied rice accessions (n = 184).

**Fig. S2.** Histograms of agro-morphological traits.

**Fig. S3.** Pearson’s correlation among yield and other agro-morphological traits in 181 *aus* rice genotypes.

**Fig. S4**. Percentages of accessions within different *aus* subgroups for different qualitative variables. LBC, leaf blade colour; LBP, leaf blade pubescence; BLSC, basal leaf sheath colour; CmA, culm angle; Cs, culm strength; FLA, flage leaf angle; CmIC, culm internode colour; CC, collar colour; EXs, panicle exertion; PnT, panicle type.

**Fig. S5**. Percentages of accessions within different *aus* subgroups for different qualitative variables. ApC, apiculus colour; AC, auricle colour; An, awning; AnC, awn colour; LmPC, lema and palea colour; LmPB, lemma and palea pubescence; SCC, seed coat colour; End, endosperm type; Thr, panicle threshability; Sen, leaf senescence.

**Fig. S6.** Quantile-Quantile plots of GWAS results using agronomic traits and principal component scores. The red line represents an ideal case where theoretical test statistic quantiles match the simulated test statistic quantiles. DtF, days to 50% flowering; Ht, plant height; Tn, tiller number; FLW, flag leaf width; PnW, panicle weight; PnL, panicle length, SpkN, spikelets per panicle; GW, 1000-grain weight; Yld, yield per plot (m^2^); HI, harvest index; PC1, principal component 1; PC2, principal component 2; PC3, principal component 3.

**Fig. S7.** Manhattan plots for individual agronomic traits. (A) Days to 50% flowering, (B) plant height, (C) Tiller number, (D) Flag leaf width, (E) Panicle length, (F) Panicle weight, (G) Spikelets per panicle, (H) 1000-grain weight, (I) Yield per plot (m^2^), (J) Harvest index. The colocalization of previously identified genes (black font) and QTLs (blue font) are shown.

**Fig. S8.** LD decay by chromosome. Insert is the distance (Kbp) where LD drops below *r^2^* of 0.1 for each chromosome.

**Fig. S9.** The box plots showing phenotypic distribution of *aus* accessions carrying the different alleles at the index SNPs (see Table 1) in the QTLs detected for PC1. (A) *qPC1-1.1,* (B) *qPC1-2.1,* (C) *qPC1-4.1,* (D) *qPC1-5.1,* (E) *qPC1-7.1,* (F), *qPC1-8.1,* (G) *qPC1-8.2.* Traits not differing significantly between two alleles are not shown.

**Fig. S10:** Box plots showing phenotypic distribution for *aus* accessions carrying the different alleles at the index SNPs in the QTLs detected for PC2. (A) *qPC2-1.1,* (B) *qPC2-7.1,* (C) *qPC2-7.2,* (D) *qPC2-11.1,* (E) *qPC2-11.2.* Traits not differing significantly between two alleles are not shown.

**Fig. S11:** Box plots showing phenotypic distribution for *aus* accessions carrying the different alleles at the index SNPs in the QTLs detected for PC3. (A) *qPC3-1.1*, (B) *qPC3-1.2*, (C) *qPC3-3.1*, (D) *qPC3-5.1*, (E) *qPC3-5.2.* Traits not differing significantly between two alleles are not shown.

**Dataset S1.** Details of 181 *aus* rice accessions used in the study.

**Dataset S2.** Agro-morphological traits recorded on 181 *aus* rice accessions.

**Dataset S3.** Principal component scores and mean values of 11 agro-morphological traits used in GWAS.

**Dataset S4.** Agronomical characteristics of *aus* subpopulations.

**Dataset S5.** Genes within *qPC2-1.1* QTL region (27.93 – 28.25 Mb).

**Dataset S6.** Genes within *qPC1-7.1* QTL region (0.73 – 1.05 Mb).


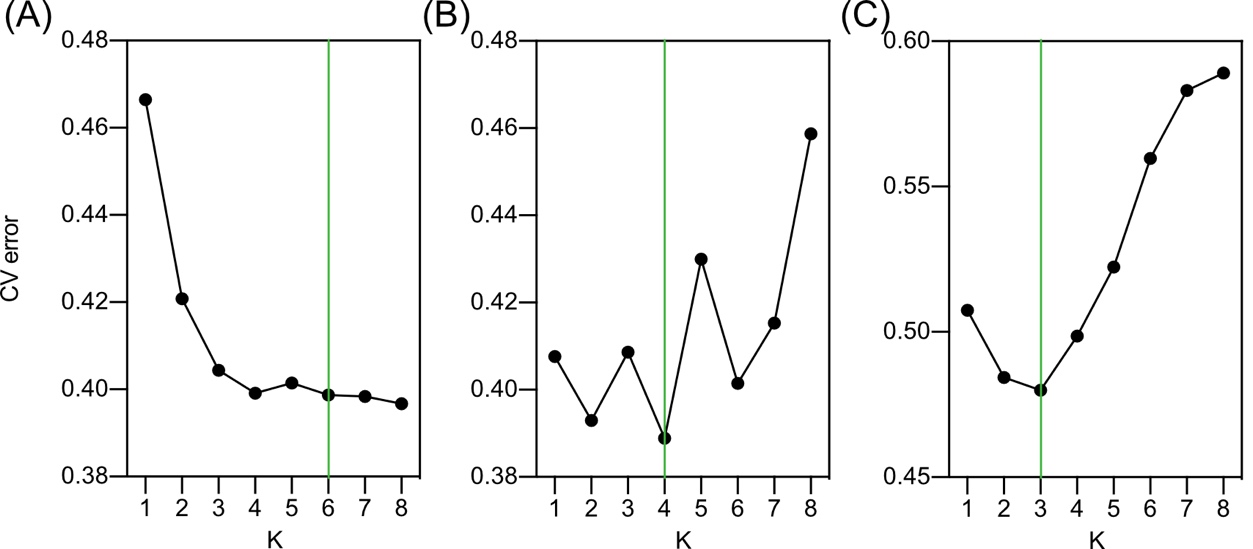


**Fig S1.** Plots of cross validation error values generated from ADMIXTURE software for (A) over all 181 *aus* accessions, (B) Bangladesh *aus* accessions and (C) Indian *aus* accessions.

**Table S1.** *F_st_* divergences between estimated *aus* subpopulations (*K* = 1-6) detected through ADMIXTURE analysis.

|  | K1 | K2 | K3 | K4 | K5 | K6 |
| --- | --- | --- | --- | --- | --- | --- |
| K1 | NA |  |  |  |  |  |
| K2 | 0.469 | NA |  |  |  |  |
| K3 | 0.235 | 0.464 | NA |  |  |  |
| K4 | 0.345 | 0.415 | 0.345 | NA |  |  |
| K5 | 0.185 | 0.518 | 0.202 | 0.396 | NA |  |
| K6 | 0.251 | 0.514 | 0.162 | 0.395 | 0.183 | NA |

Abbreviation: NA, not applicable

**Table S2.** Descriptive statistics of quantitative traits in the studied rice accessions (n = 184).

| **Trait** | **Mean** | **SD** | **Min** | **Max** | **Skewness** | **Kurtosis** | **CV%** | **GCV** | **PCV** | **hBS** |
| --- | --- | --- | --- | --- | --- | --- | --- | --- | --- | --- |
| DtF | 72.34 | 11.5 | 53.4 | 109.4 | 1.03 ** | 3.84 * | 2.72 | 15.64 | 15.88 | 96.99 |
| Ht | 128.58 | 18.28 | 83.08 | 187.48 | 0.73 ** | 3.81 * | 4.54 | 13.92 | 14.61 | 90.86 |
| Tn | 13.19 | 4.94 | 2.63 | 33.74 | 0.79 ** | 4.12 * | 20.02 | 29.64 | 35.62 | 69.23 |
| FLL | 39 | 6.84 | 17.75 | 72.09 | 0.63 ** | 5.86 ** | 11.25 | 14.13 | 17.99 | 61.69 |
| FLW | 1.47 | 0.43 | 0.37 | 2.64 | 0.28 ^ns^ | 3.1 ^ns^ | 14.88 | 24.61 | 28.65 | 73.81 |
| PnW | 2.09 | 0.93 | 0.21 | 5.67 | 0.91 ** | 4.44 ** | 18.14 | 37.49 | 42.1 | 79.32 |
| PnL | 22.48 | 2.57 | 16.22 | 33.51 | 0.69 ** | 4.91 ** | 5 | 9.18 | 10.43 | 77.39 |
| SpkN | 112.81 | 50.05 | 31.57 | 341.97 | 1.27 ** | 5.49 ** | 9.06 | 43.53 | 44.53 | 95.58 |
| GW | 24.5 | 4.39 | 11.68 | 35.75 | -0.2 ^ns^ | 3.39 ^ns^ | 2.88 | 17.67 | 17.91 | 97.29 |
| Yld | 759.91 | 267.25 | 106.6 | 1403.41 | 0.13 ^ns^ | 2.79 ^ns^ | 27.62 | 10.16 | 32.17 | 9.98 |
| HI | 0.43 | 0.11 | 0.07 | 0.65 | -0.63 ** | 3.52 ^ns^ | 17.75 | 15.67 | 24.51 | 40.86 |

Abbreviation:

DtF, days to 50% flowering; Ht, plant height; Tn, tiller number; FLL, flag leaf length; FLW, flag leaf width; PnW, panicle weight; PnL, panicle length, SpkN, spikelets per panicle; GW, 1000-grain weight; Yld, Yield per plot (m^2^); HI, harvest index; SD, standard deviation; Min, minimum; Max, maximum; CV, coefficient of variation; GCV, genotypic coefficient of variation; PCV, phenotypic coefficient of variation; hBS, broad sense heritability

*, **, significant at 5%, and 1% level of significance, respectively; ^ns^, not significant


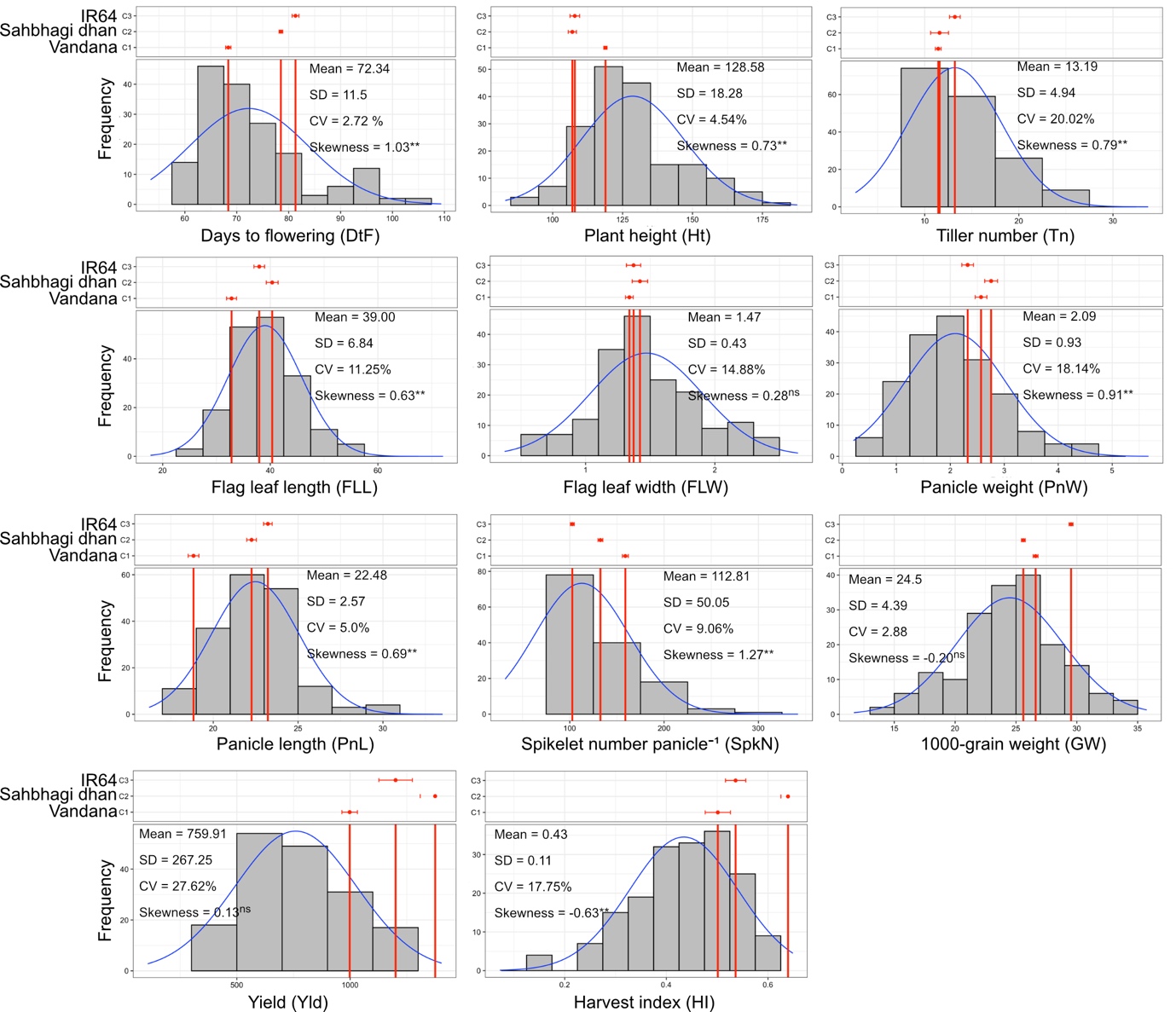


**Fig. S2.** Histograms of agro-morphological traits.

DtF, days to 50% flowering; Ht, plant height; Tn, tiller number; FLL, flag leaf length; FLW, flag leaf width; PnW, panicle weight; PnL, panicle length, SpkN, spikelets per panicle; GW, 1000-grain weight; Yld, Yield per plot (m^2^); HI, harvest index.


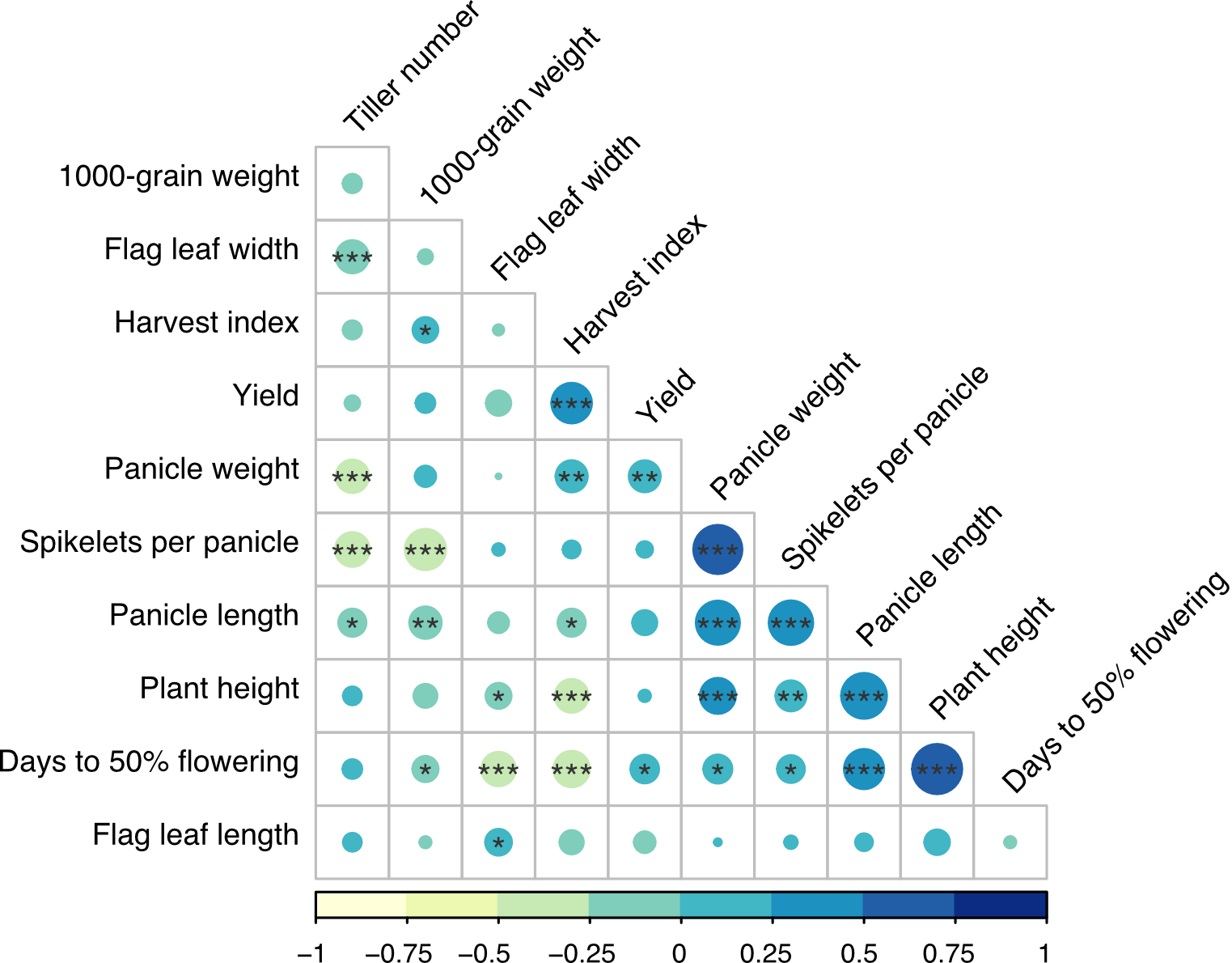


**Fig. S3.** Pearson’s correlation among yield and other agro-morphological traits in 181 *aus* rice genotypes.


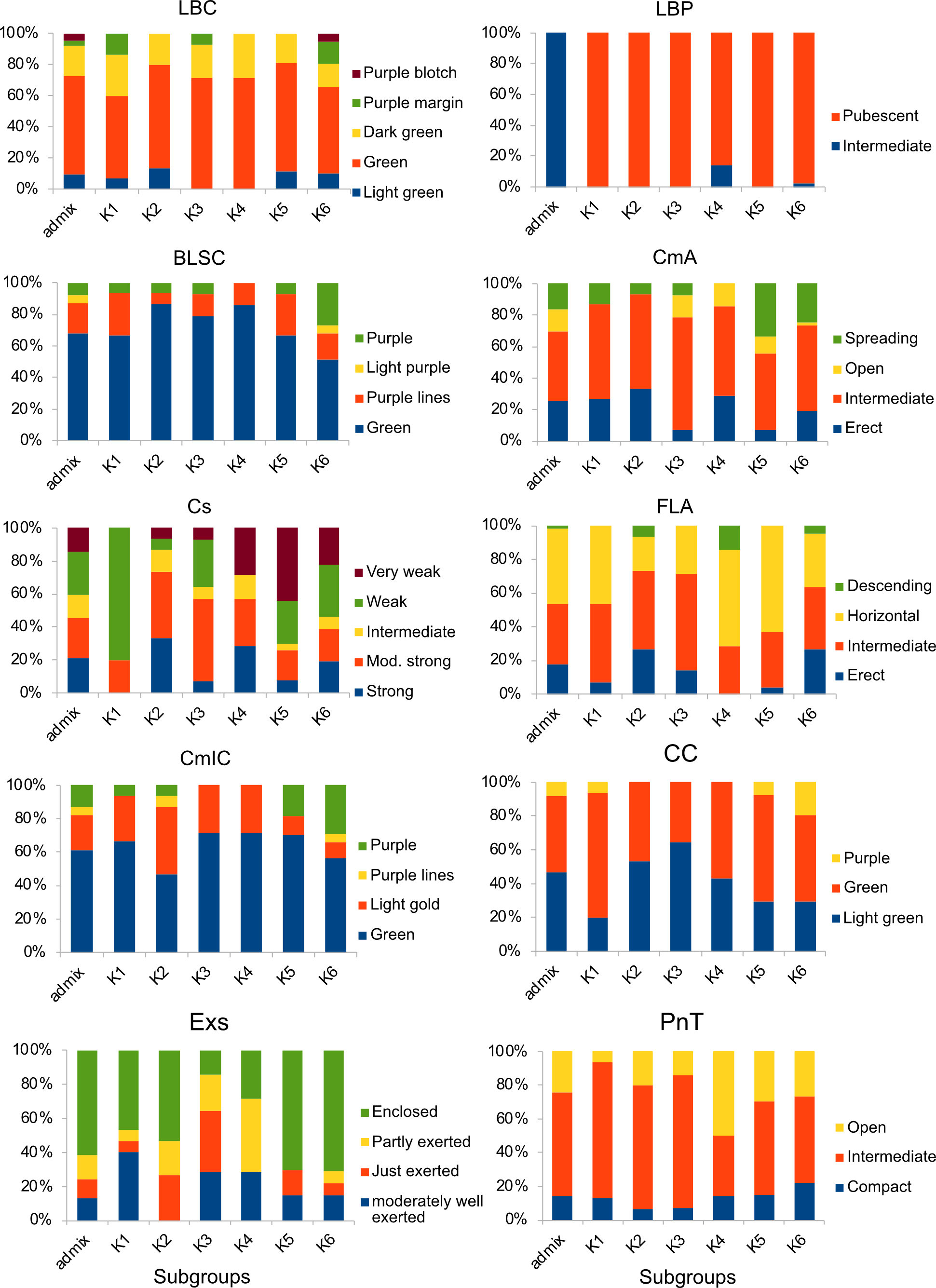


**Fig. S4**. Percentages of accessions within different *aus* subgroups for different qualitative variables.

LBC, leaf blade colour; LBP, leaf blade pubescence; BLSC, basal leaf sheath colour; CmA, culm angle; Cs, culm strength; FLA, flage leaf angle; CmIC, culm internode colour; CC, collar colour; EXs, panicle exertion; PnT, panicle type.


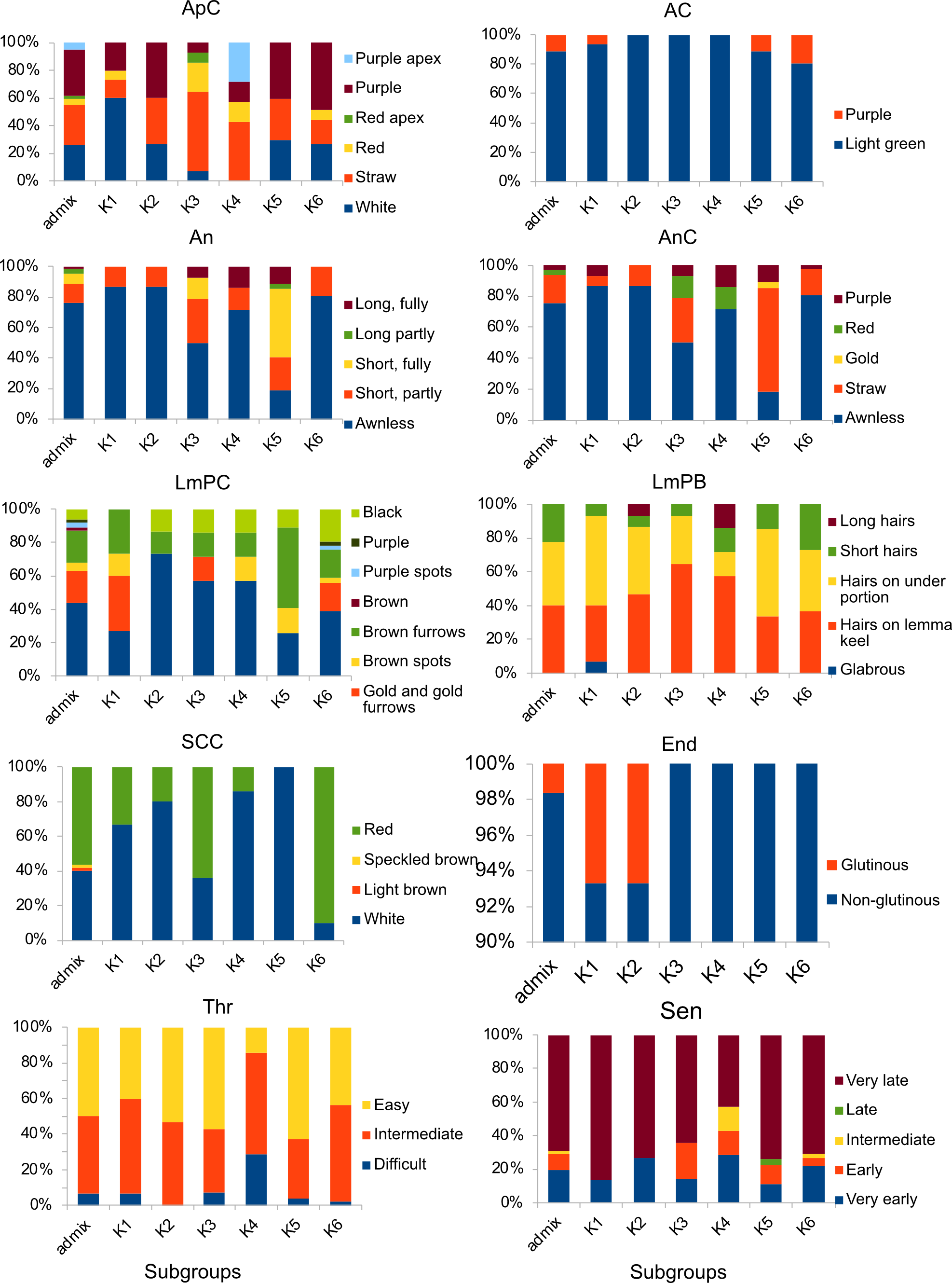


**Fig. S5**. Percentages of accessions within different *aus* subgroups for different qualitative variables.

ApC, apiculus colour; AC, auricle colour; An, awning; AnC, awn colour; LmPC, lema and palea colour; LmPB, lemma and palea pubescence; SCC, seed coat colour; End, endosperm type; Thr, panicle threshability; Sen, leaf senescence.

**
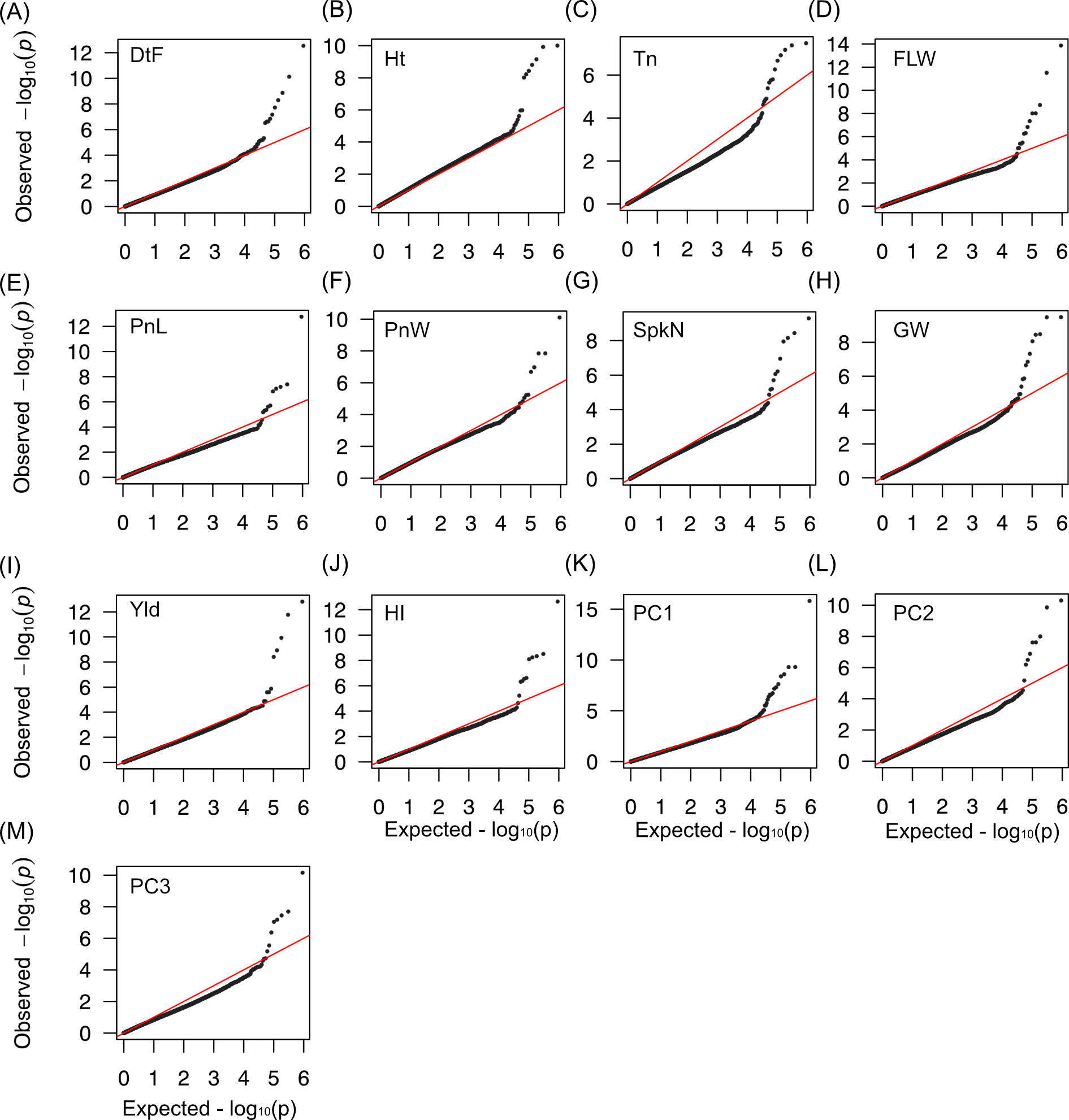
**

**Fig. S6.** Quantile-Quantile plots of GWAS results using agronomic traits and principal component scores. The red line represents an ideal case where theoretical test statistic quantiles match the simulated test statistic quantiles.

DtF, days to 50% flowering; Ht, plant height; Tn, tiller number; FLW, flag leaf width; PnW, panicle weight; PnL, panicle length, SpkN, spikelets per panicle; GW, 1000-grain weight; Yld, yield per plot (m^2^); HI, harvest index; PC1, principal component 1; PC2, principal component 2; PC3, principal component 3.


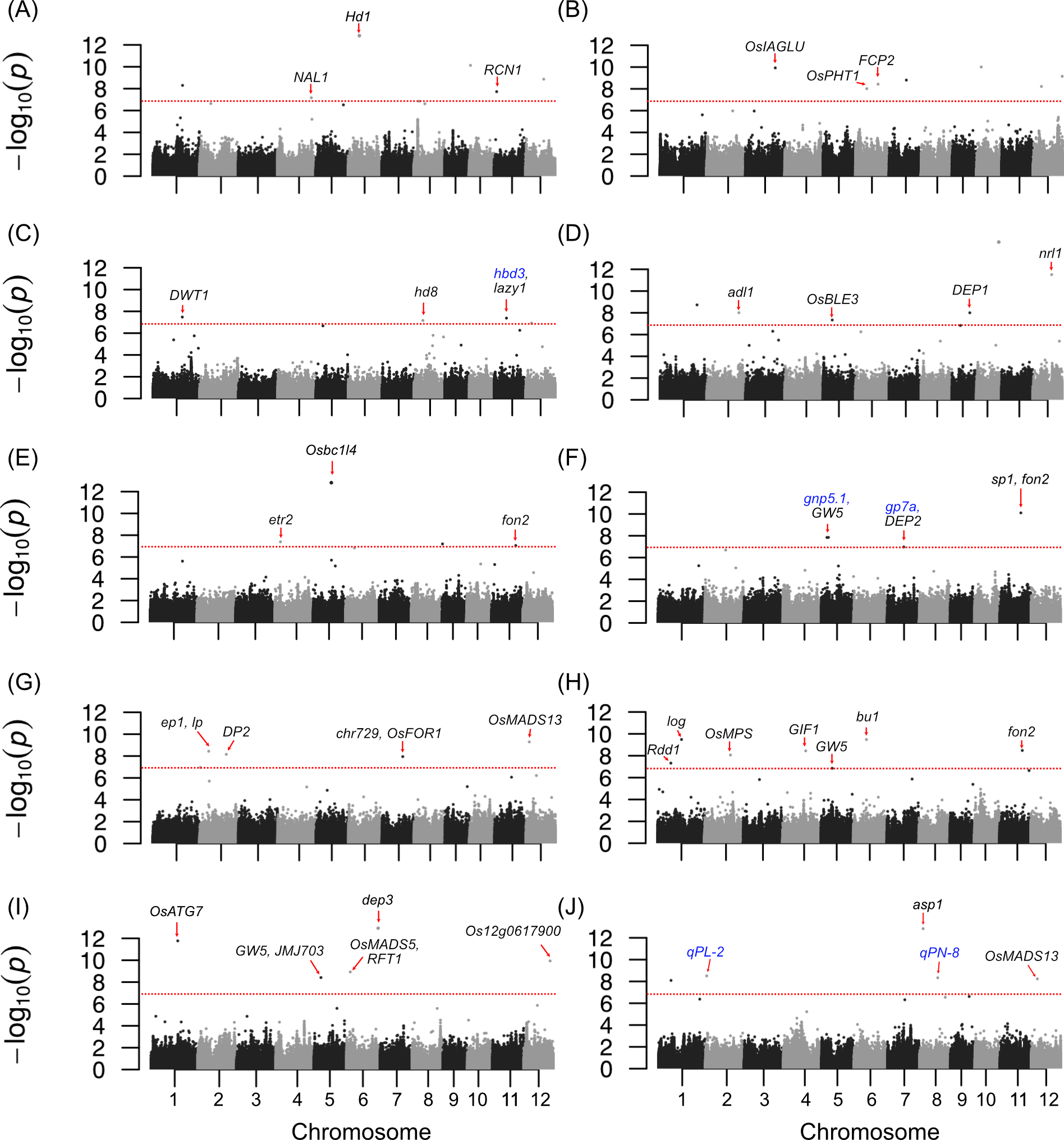


**Fig. S7.** Manhattan plots for individual agronomic traits. (A) Days to 50% flowering, (B) plant height, (C) Tiller number, (D) Flag leaf width, (E) Panicle length, (F) Panicle weight, (G) Spikelets per panicle, (H) 1000-grain weight, (I) Yield per plot (m^2^), (J) Harvest index. The colocalization of previously identified genes (black font) and QTLs (blue font) are shown.


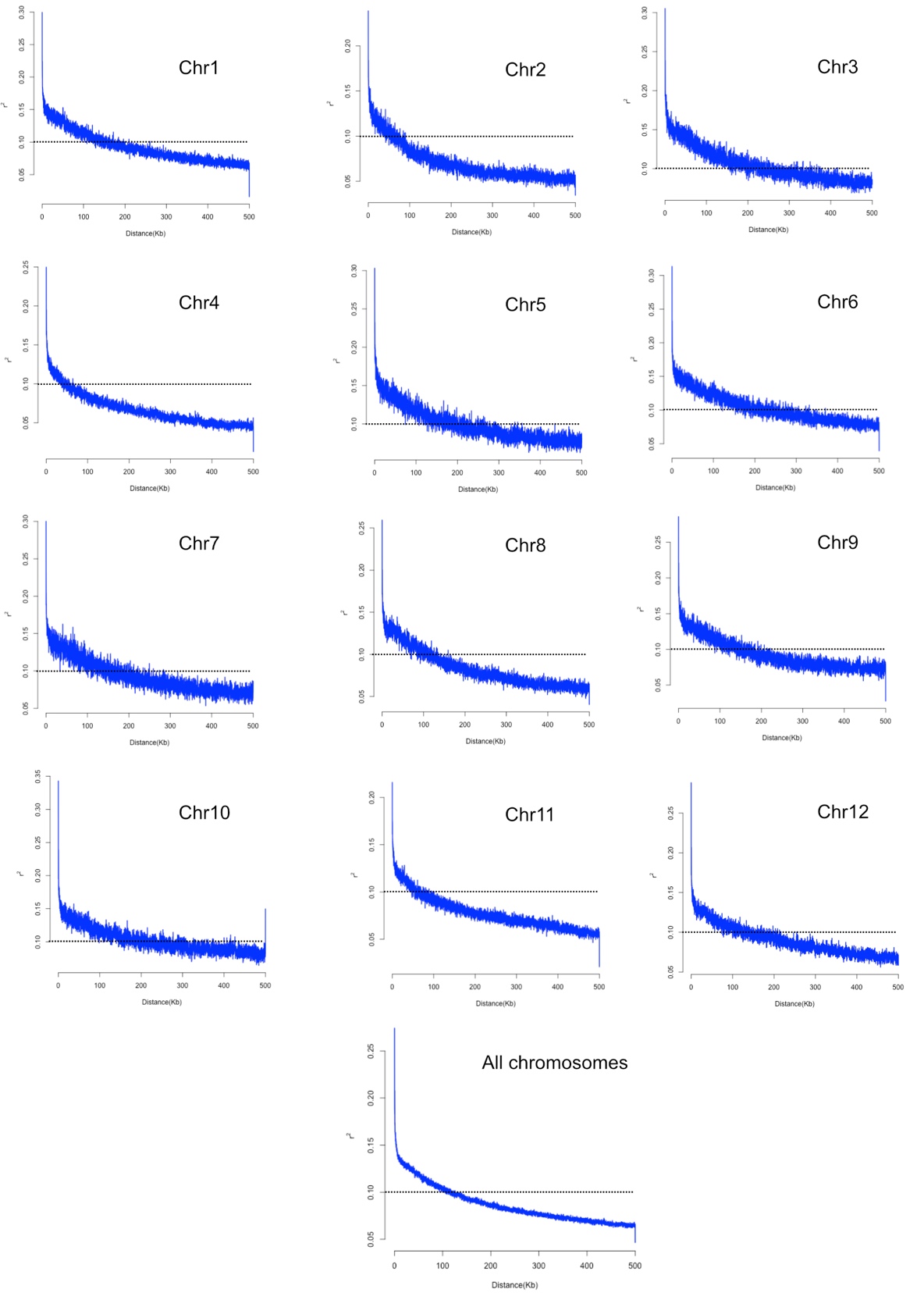


**Fig. S8.** LD decay by chromosome. Insert is the distance (Kbp) where LD drops below *r^2^* of 0.1 for each chromosome.


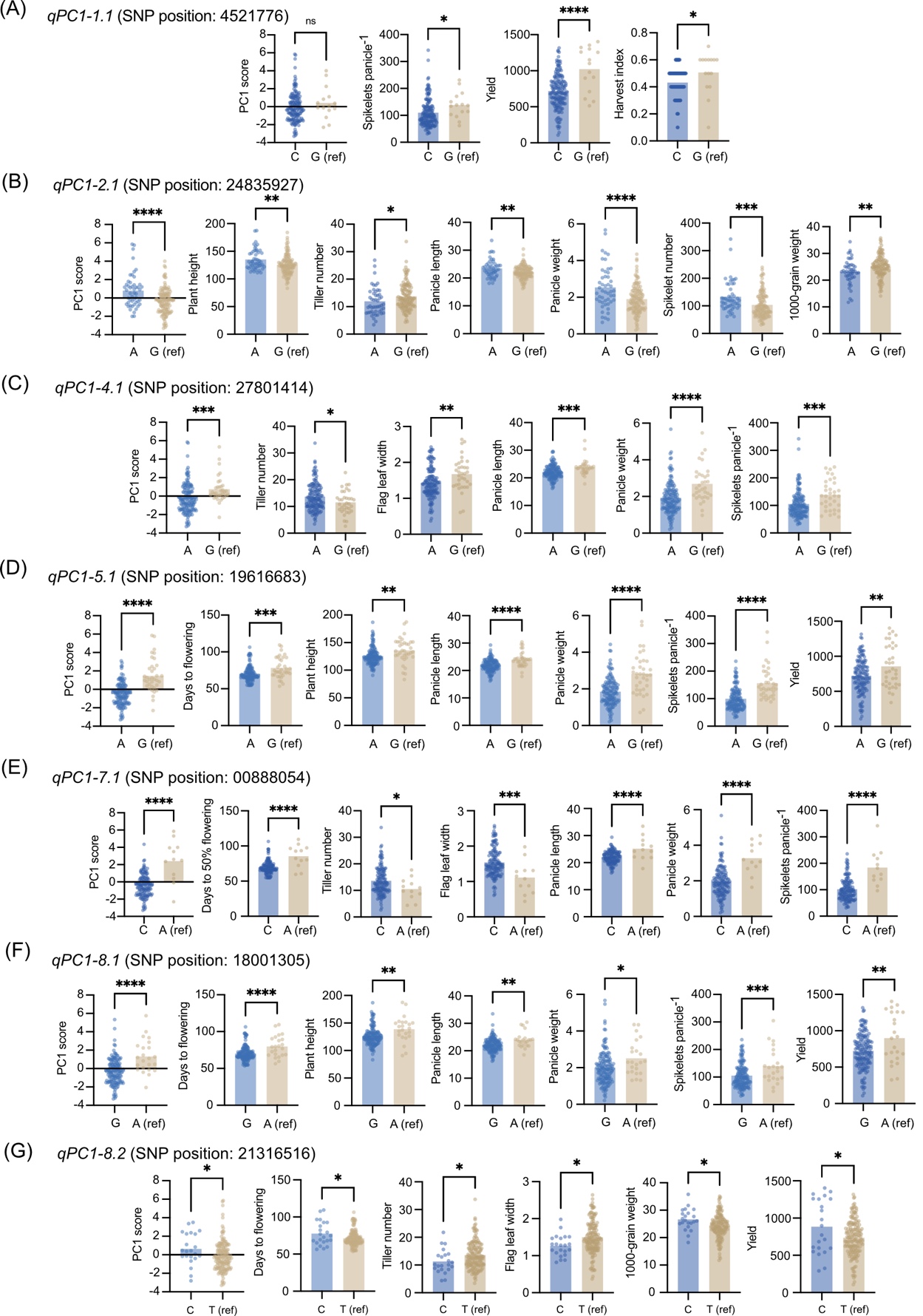


**Fig. S9.** The box plots showing phenotypic distribution of *aus* accessions carrying the different alleles at the index SNPs (see Table 1) in the QTLs detected for PC1. (A) *qPC1-1.1,* (B) *qPC1-2.1,* (C) *qPC1-4.1,* (D) *qPC1-5.1,* (E) *qPC1-7.1,* (F), *qPC1-8.1,* (G) *qPC1-8.2.* Traits not differing significantly between two alleles are not shown.


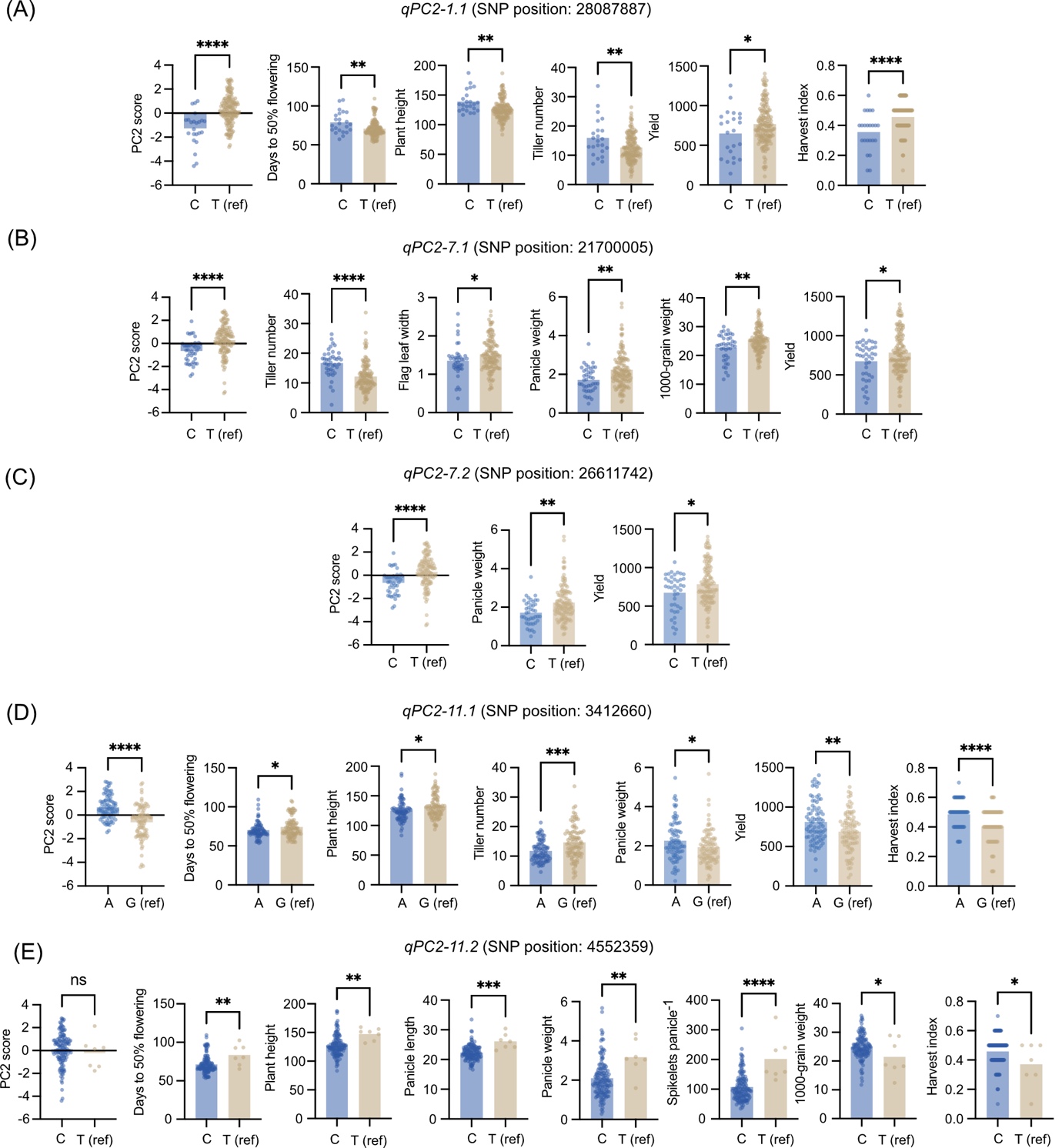


**Fig. S10:** Box plots showing phenotypic distribution for *aus* accessions carrying the different alleles at the index SNPs in the QTLs detected for PC2. (A) *qPC2-1.1,* (B) *qPC2-7.1,* (C) *qPC2-7.2,* (D) *qPC2-11.1,* (E) *qPC2-11.2.* Traits not differing significantly between two alleles are not shown.


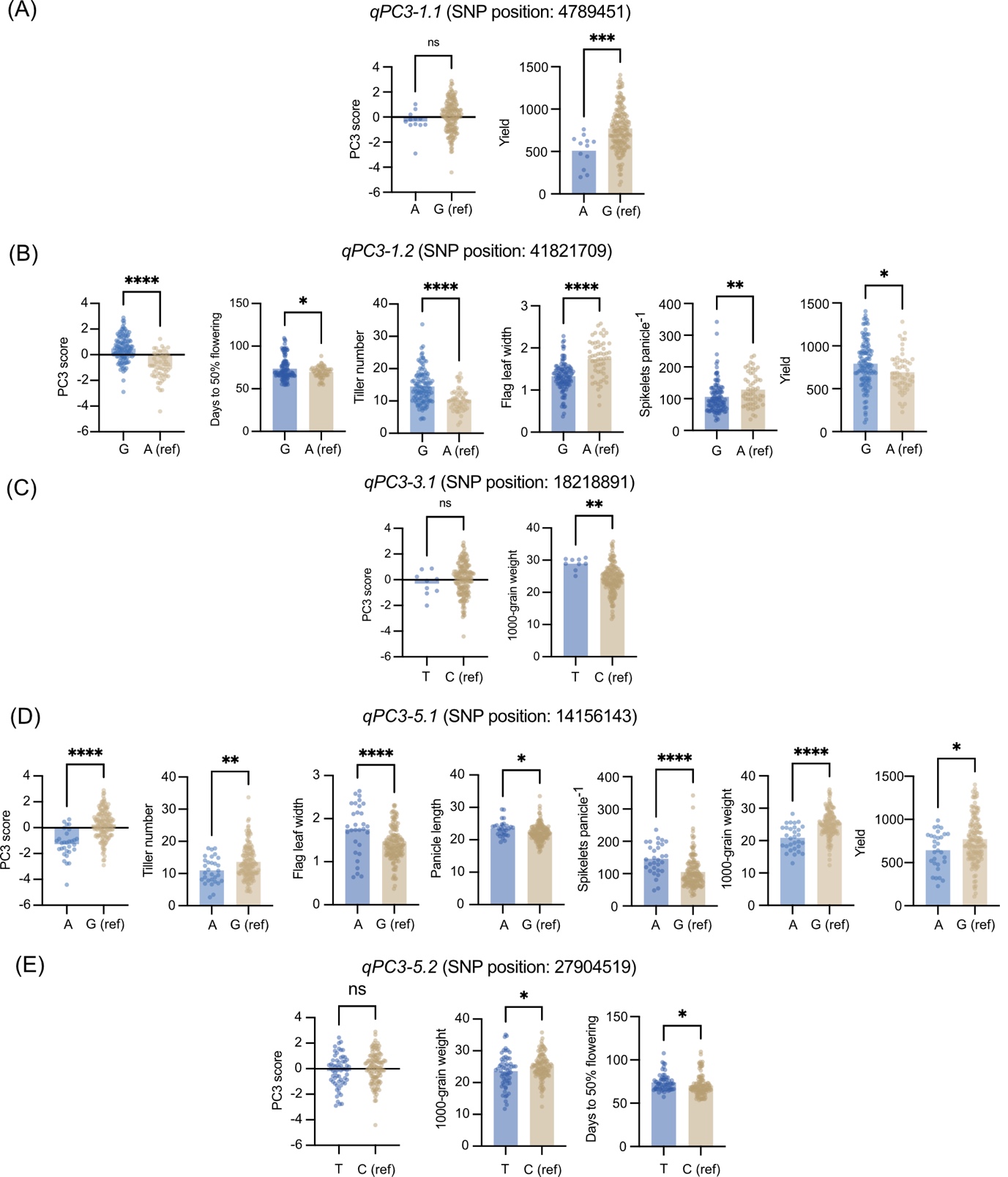


**Fig. S11:** Box plots showing phenotypic distribution for *aus* accessions carrying the different alleles at the index SNPs in the QTLs detected for PC3. (A) *qPC3-1.1*, (B) *qPC3-1.2*, (C) *qPC3-3.1*, (D) *qPC3-5.1*, (E) *qPC3-5.2.* Traits not differing significantly between two alleles are not shown.
